# An integrated single-cell reference atlas of the human endometrium

**DOI:** 10.1038/s41588-024-01873-w

**Published:** 2024-08-28

**Authors:** Magda Marečková, Luz Garcia-Alonso, Marie Moullet, Valentina Lorenzi, Robert Petryszak, Carmen Sancho-Serra, Agnes Oszlanczi, Cecilia Icoresi Mazzeo, Frederick C. K. Wong, Iva Kelava, Sophie Hoffman, Michał Krassowski, Kurtis Garbutt, Kezia Gaitskell, Slaveya Yancheva, Ee Von Woon, Victoria Male, Ingrid Granne, Karin Hellner, Krishnaa T. Mahbubani, Kourosh Saeb-Parsy, Mohammad Lotfollahi, Elena Prigmore, Jennifer Southcombe, Rebecca A. Dragovic, Christian M. Becker, Krina T. Zondervan, Roser Vento-Tormo

**Affiliations:** 1 Wellcome Sanger Institute, Cambridge, UK; 2 Oxford Endometriosis Care Centre, Nuffield Department of Women’s & Reproductive Health, University of Oxford, Oxford, UK; 3 European Bioinformatics Institute-European Molecular Biology Laboratory, Cambridge, UK; 4 Centre for Human Genetics, University of Oxford, Oxford, UK; 5 Nuffield Division of Clinical Laboratory Sciences, Radcliffe Department of Medicine, University of Oxford, Oxford, UK; 6 Department of Cellular Pathology, John Radcliffe Hospital, Oxford, UK; 7 Department of Metabolism, Digestion and Reproduction, Institute of Reproductive and Developmental Biology, Imperial College London, London, UK; 8 The Fertility Centre, Chelsea and Westminster Hospital, London, UK; 9 Department of Haematology, University of Cambridge, Cambridge, UK; 10 Cambridge Biorepository for Translational Medicine (CBTM), NIHR Cambridge Biomedical Research Centre, Cambridge, UK; 11 Department of Surgery, University of Cambridge, Cambridge, UK; 12 Wellcome–MRC Cambridge Stem Cell Institute, University of Cambridge, Cambridge, UK

**Keywords:** RNA sequencing, Transcriptomics

## Abstract

The complex and dynamic cellular composition of the human endometrium remains poorly understood. Previous endometrial single-cell atlases profiled few donors and lacked consensus in defining cell types. We introduce the Human Endometrial Cell Atlas (HECA), a high-resolution single-cell reference atlas (313,527 cells) combining published and new endometrial single-cell transcriptomics datasets of 63 women with and without endometriosis. HECA assigns consensus and identifies previously unreported cell types, mapped in situ using spatial transcriptomics and validated using a new independent single-nuclei dataset (312,246 nuclei, 63 donors). In the functionalis, we identify intricate stromal–epithelial cell coordination via transforming growth factor beta (TGFβ) signaling. In the basalis, we define signaling between fibroblasts and an epithelial population expressing progenitor markers. Integration of HECA with large-scale endometriosis genome-wide association study data pinpoints decidualized stromal cells and macrophages as most likely dysregulated in endometriosis. The HECA is a valuable resource for studying endometrial physiology and disorders, and for guiding microphysiological in vitro systems development.

## Abstract (web summary)

The Human Endometrial Cell Atlas integrates single-cell transcriptomic datasets from women with and without endometriosis. Novel and known cell types are registered using spatial transcriptomics to provide a comprehensive map of the human endometrium in controls and endometriosis cases.

## Main

Human reproduction depends on the endometrium, the inner mucosal lining of the uterus. It prepares an optimal environment for embryo implantation and development. In the absence of pregnancy, the endometrium sheds each month during menstruation. Morphologically, the endometrium is composed of two layers: the ever-changing functionalis (adjacent to the uterine cavity) and the relatively constant basalis (adjacent to the myometrium). In response to ovarian steroid hormones, the functionalis undergoes repeated cycles of shedding, repair without scarring, extensive growth and differentiation^1,2^.

At the cellular level, the endometrium is particularly complex. Its epithelium consists of a horizontally interconnected network of basalis glands^3–5^ contiguous with coiled functionalis glands extending vertically towards the uterine cavity, where a layer of functionalis luminal cells lines the endometrial surface. The basalis glands harbor epithelial stem/progenitor cells needed to regenerate the functionalis glands after menstruation^6–10^. Stromal, fibroblast, perivascular (PV) and endothelial cells provide support and structural integrity, including rich vasculature within the tissue. An array of immune cells play crucial roles in endometrial shedding, repair^11,12^ and embryo implantation^13^. Fine-tuned and timely communication between these cells is key for endometrial functioning and menstrual cycle progression.

During reproductive years, the endometrium is highly heterogeneous, both inter- and intra-individually, requiring a large sample size to account for the dynamic changes it undergoes both in time (across the menstrual cycle) and in space (across different tissue microenvironments). Several foundational studies atlasing the cellular composition of the human endometrium with single-cell^14–21^ and spatial^15–17^ technologies have been published. However, these cell censuses so far profiled a limited number of samples, and lacked even coverage of the menstrual cycle phases, consensus cell state annotation and reproducible marker gene signatures. Additionally, they varied considerably in terms of clinical and phenotypic characterization of the individuals profiled. These factors have complicated comparisons across studies, with, for example, inconsistencies in the identification and naming of epithelial and stromal cell states. An integrated single-cell reference atlas of the human endometrium, encompassing the widest possible range of cell states and samples, is now warranted.

Endometrial heterogeneity is further increased by endometrial/uterine disorders which are highly prevalent globally^22–24^. For example, ~190 million women world-wide suffer from endometriosis^22–24^, where endometrial-like cells grow outside of the uterus (that is, ectopically). Conflicting evidence exists about whether and to what extent the endometrium itself (that is, the eutopic endometrium) differs between those with and without endometriosis^25,26^. Recently, single-cell studies, analyzing small sample sizes, reported dysregulation of the stromal and immune compartments in the endometrium of women with endometriosis to various degrees^16,18,20,27,28^. Larger sample sets are now needed to unpick whether and how the endometrium differs in those with and without endometriosis. Well-annotated reference cell atlases can provide invaluable insights.

Here, we assemble a consensus HECA (https://www.reproductivecellatlas.org/endometrium_reference.html) by harmonizing the transcriptomic and donor metadata information of ~626,000 cells and nuclei from previously published and newly generated datasets. We identify cell populations not reported by previous atlases, including an epithelial *CDH2*^*+*^ population in the basalis and distinct populations of functionalis epithelial and stromal cells characteristic of the early secretory phase. We describe the molecular signals likely mediating the spatiotemporal organization and function of cellular niches across the menstrual cycle and provide an interactive portal to visualize and query the predicted cell–cell communication at https://www.reproductivecellatlas.org/endometrium_reference.html. Finally, we use the HECA to give cellular context to genetic associations identified by the largest endometriosis genome-wide association study (GWAS) meta-analysis^29^. This analysis identifies macrophages and subsets of decidualized stromal cells as the cell types expressing the genes affected by the variants associated with endometriosis.

## Results

### Harmonized data to generate the HECA

**Fig. 1:**
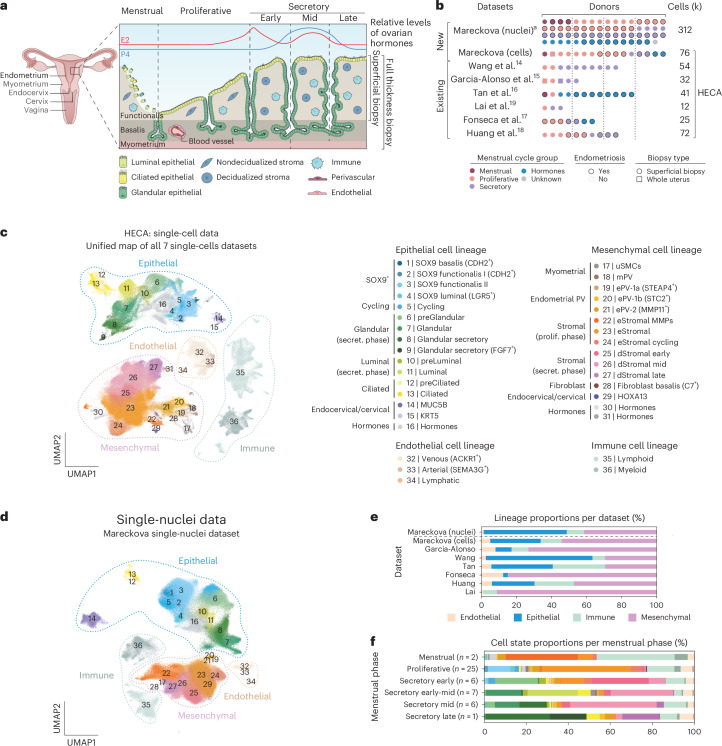
Harmonized cellular map of the human endometrium. **a**, Schematic illustration of the human uterus and cellular composition of the endometrium as it undergoes morphological changes across the menstrual cycle. **b**, List of datasets analyzed and contribution of the number of donors, cells/nuclei, endometrial histology and endometriosis status of all samples profiled per dataset. **c**, UMAP projections of the HECA scRNA-seq data from a total of 63 individuals and 313,527 cells colored by cell state. **d**, UMAP projections of snRNA-seq data from a total of 63 individuals and 312,246 nuclei colored by cell state. Dot colour corresponds to cell states described in **c**. **e**, Bar plot showing the contribution of each of the scRNA-seq datasets to the main cellular lineages (endothelial, epithelial, immune and mesenchymal lineages) as shown in **c**. **f**, Bar plot showing the cellular composition of a total of 47 endometrial biopsies from the menstrual (*n* = 2), proliferative (*n* = 25), early secretory (*n* = 6), early/mid secretory (*n* = 7), mid secretory (*n* = 6) and late secretory (*n* = 1) phases of the menstrual cycle for the scRNA-seq data presented in **c**. Biopsies from donors on hormones (*n* = 14) and samples assigned as secretory phase without available subcategorisation into early/mid/late secretory (*n* = 2) are shown in Extended Data Fig. 1d. Bar colour corresponds to cell states described in **c**. ^a^Five donors are shared between Mareckova scRNA-seq and snRNA-seq datasets. ePV, endometrial PV cells; mPV, myometrial PV cells; prolif., proliferative; secret., secretory.

**Extended Data Fig. 1:**
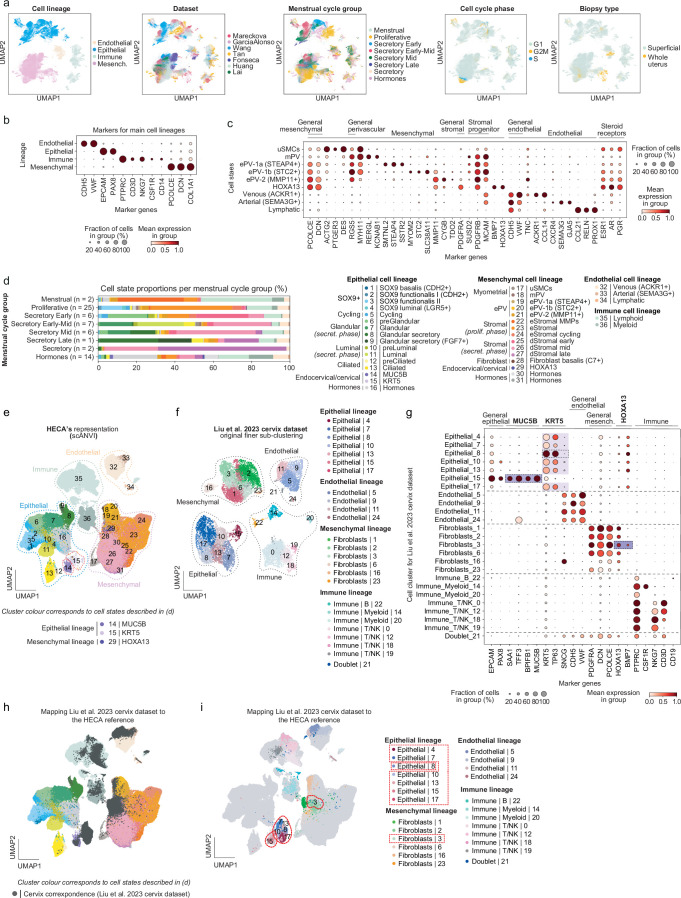
Single-cell RNA-sequencing datasets of the Human Endometrial Cell Atlas (HECA) and the cervix. **a**, UMAP projections of scRNA-seq data for HECA coloured by cell lineage, dataset, menstrual cycle, cell cycle phase and biopsy type. **b**, Dot plot showing normalised, log-transformed and variance-scaled expression of genes (x-axis) characteristic of the main cell lineages (y-axis) in the HECA. **c**, Dot plot showing normalised, log-transformed and variance-scaled expression of genes (x-axis) characteristic of mesenchymal and endothelial cells (y-axis) in the HECA. **d**, Bar plot showing the cellular composition of endometrial biopsies in the different menstrual cycle groups (y-axis). **e**, UMAP projection of a scANVI representation of the HECA coloured by the cell states identified. Red dotted-lined shapes outline the MUC5B, KRT5 and HOXA13 populations. **f**, UMAP projection of the Liu et al.^30^ scRNA-seq dataset of human cervix coloured by Liu’s clusters and the four main cell lineages. **g**, Dot plot showing normalised, log-transformed and variance-scaled expression of genes (x-axis) characteristic of the cell clusters identified in the Liu et al.^30^ cervix dataset by the authors (y-axis). Purple rectangles highlight the epithelial and mesenchymal clusters that expressed markers characteristic of the MUC5B, KRT5 and HOXA13 populations defined in the HECA. **h**, UMAP projection of the mapping of the Liu et al. ^30^ cervix dataset onto the scANVI representation of the HECA coloured by either the endometrial cell states identified in the HECA or the cervix cell states Liu et al.^30^ (grey). Red dotted-lined shapes outline the MUC5B, KRT5 and HOXA13 populations of the HECA. **i**, UMAP projection of the mapping of the Liu et al.^30^ cervix dataset onto the scANVI representation of the HECA coloured by the matched cervix cell clusters identified by Liu et al.^30^. Red dotted-lines outline the MUC5B, KRT5 and HOXA13 populations of the HECA. dStromal, decidualised stromal cells; ePV, endometrial perivascular cells; eStromal, endometrial stromal cells specific to proliferative phase; HECA, human endometrial cell atlas; MMPs, matrix metalloproteinases; NK, natural killer cells; scRNA-seq, single-cell RNA-sequencing; scANVI, single-cell ANnotation using Variational Inference; T, T cells; UMAP, uniform manifold approximation and projection; uSMCs, uterine smooth muscle cells.

**Extended Data Fig. 2:**
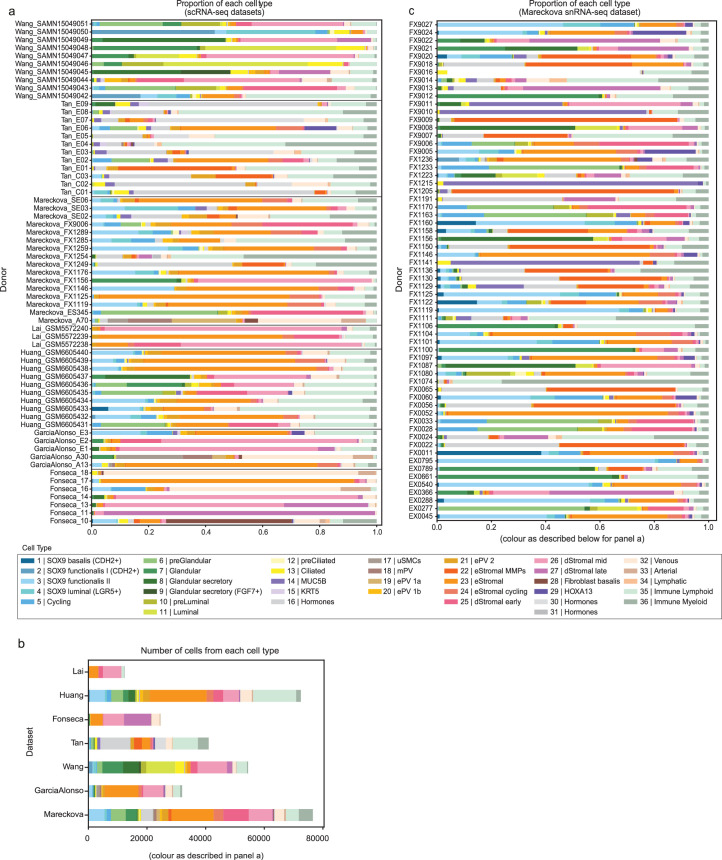
Distribution of cell types across samples in cells and nuclei. **a**, Bar plot showing the proportion of cell types in each sample. Each row corresponds to a donor, grouped by study and coloured by cell type. **b**, Bar plot representing the number of cells of each cell type in each dataset. Each row represents a dataset, coloured by cell type. **c**, Bar plot showing the proportion of cell types in each donor from the nuclei dataset. Each row represents a donor, coloured by cell type.

**Extended Data Fig. 3:**
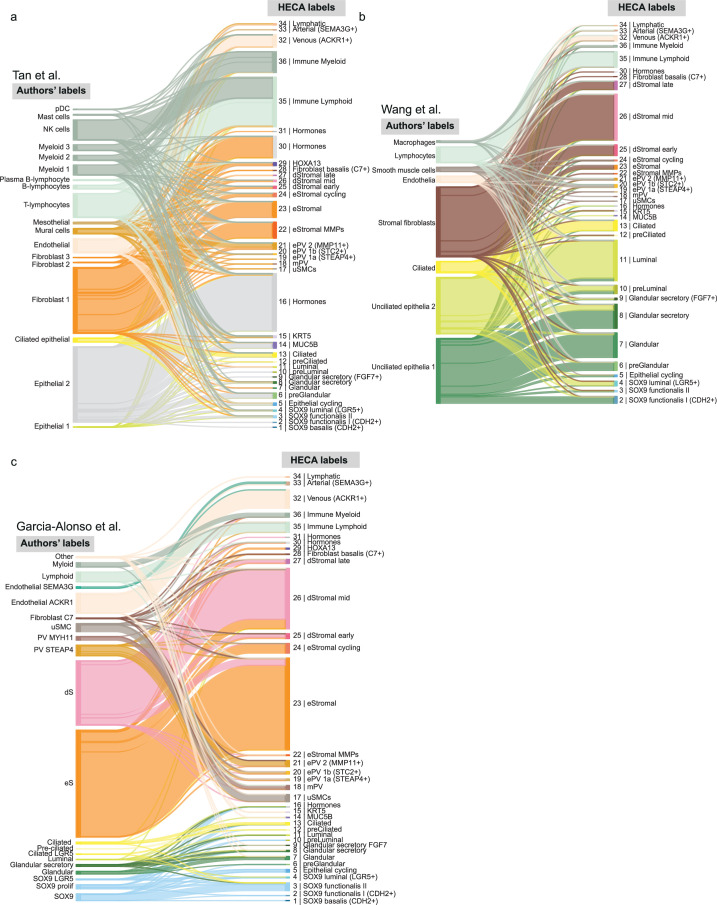
Comparison of cell type labels from original publications and HECA. Sankey plot showing the correspondence between cell type labels from original publications (left) and HECA (right) from the Tan (**a**), Wang (**b**) and Garcia-Alonso (**c**) datasets respectively. In each plot, the width of each line is proportional to number of cells.

To comprehensively define endometrial cell types and states across the menstrual cycle, we analyzed a total of ~626,000 high-quality cells and nuclei from 121 individuals (Fig. 1a,b and Supplementary Note 1). First, we created a single-cell reference atlas (that is, the HECA; Fig. 1c), by integrating six publicly available single-cell RNA sequencing (scRNA-seq) datasets (Wang et al.^14^, Garcia-Alonso et al.^15^, Tan et al.^16^, Lai et al.^19^, Fonseca et al.^17^ and Huang et al.^18^) with our newly generated anchor dataset (termed the Mareckova (cells) dataset). The anchor dataset contained samples from donors with similar clinical characteristics as the donors profiled in the previously published datasets, allowing us to correct for dataset-specific signatures while preserving biological ones during integration (Fig. 1b and Supplementary Note 1). Harmonization of metadata across the studies and application of strict data quality control (QC) filters was essential for the integration (Methods). The final integrated HECA consisted of ~313,527 high-quality cells from seven datasets, of which ~76,000 cells were newly profiled by us (Supplementary Tables 1 and 2). It included a total of 63 individuals both with endometriosis (that is, cases) and without endometriosis (that is, controls), with samples collected either during natural cycles or when taking exogenous hormones (Fig. 1b,c, Extended Data Figs. 1a–i, 2a,b and 3 and Supplementary Table 1). Most samples analyzed were superficial biopsies of the endometrium, predominantly sampling the functionalis layer from living donors. Three samples from the uteri of donors who died of nongynecological causes contained full thickness endometrium, encompassing both the functionalis and basalis layers, with attached subjacent myometrium.

**Extended Data Fig. 4:**
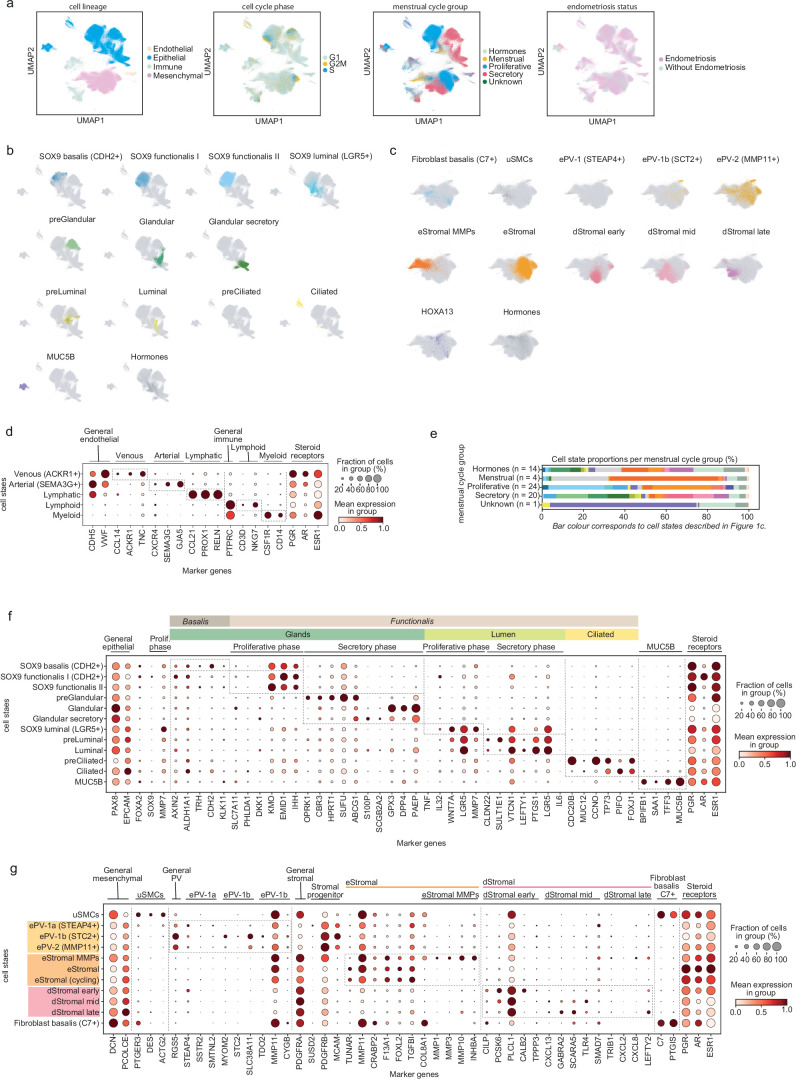
Single-nucleus RNA-sequencing cell state identification and marker gene expression. **a**, UMAP projections of the snRNA-seq data coloured by cell lineage, cell cycle phase, menstrual cycle group, and endometriosis status. **b**, UMAP projections of the epithelial cell lineage of the snRNA-seq dataset coloured by the identified epithelial cell states of the HECA as assigned by label transfer. **c**, UMAP projections of the mesenchymal cell lineage of the snRNA-seq dataset coloured by the identified mesenchymal cell states of the HECA as assigned by label transfer. **d**, Dot plot showing normalised, log-transformed and variance-scaled expression of genes (x-axis) characteristic of the endothelial and immune nuclei (y-axis). **e**, Bar plot showing the cellular composition of endometrial biopsies belonging to the different menstrual cycle groups (y-axis). **f**, Dot plot showing normalised, log-transformed and variance-scaled expression of genes (x-axis) characteristic of the identified epithelial cell states (y-axis) in snRNA-seq data. **g**, Dot plot showing normalised, log-transformed and variance-scaled expression of genes (x-axis) characteristic of the identified mesenchymal cell states (y-axis) in snRNA-seq data. dStromal, decidualised stromal cells; ePV, endometrial perivascular cells; eStromal, endometrial stromal cells specific to proliferative phase; HECA, human endometrial cell atlas; MMPs, matrix metalloproteinases; mPV, myometrial perivascular cells; Prolif., proliferative; secret., secretory; snRNA-seq, single-nucleus RNA-sequencing; UMAP, uniform manifold approximation and projection; uSMCs, uterine smooth muscle cells.

We observed striking differences between the cellular composition of the integrated scRNA-seq datasets, with variable recovery of epithelial, mesenchymal, endothelial and immune cells (Fig. 1e and Supplementary Table 3). Choice of tissue digestion protocol, sampling bias (technical variation), menstrual cycle stage and use of exogenous hormones (biological variation) could all be responsible for the differences observed (Supplementary Note 1 and Extended Data Fig. 1a–i). The dataset-specific cellular proportions prompted us to generate an independent single-nucleus RNA sequencing (snRNA-seq) dataset for 63 additional donors (Fig. 1b,d), five of them overlapping with the scRNA-seq dataset. The large number of individuals in the snRNA-seq dataset allowed us to overcome the technical variation introduced when data are generated by different laboratories. We profiled ~312,246 high-quality nuclei from snap-frozen samples of superficial endometrial biopsies (Fig. 1b,d, Extended Data Figs. 2c and 4a and Supplementary Table 2), collected during natural cycles and when taking exogenous hormones, and including donors with and without endometriosis (Fig. 1b). This dataset represents the largest set of human endometrial samples profiled at the single-cell/single-nucleus transcriptomic level by a single laboratory so far. To align the cell state annotations across the scRNA-seq and snRNA-seq datasets, and to determine the robustness of the HECA, we transferred cell state labels between datasets using machine learning (Methods). Of the endometrial cells identified by scRNA-seq, the majority were validated in the nuclei dataset (Extended Data Fig. 4b,c).

**Extended Data Fig. 5:**
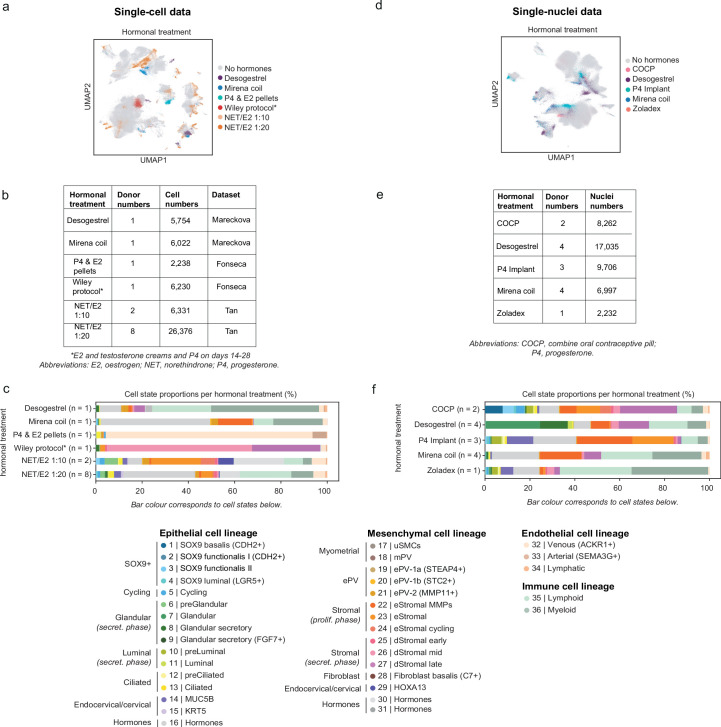
Cellular heterogeneity of samples from donors taking exogenous hormones in scRNA-seq and snRNA-seq data. **a**, UMAP projections of the scRNA-seq data coloured by hormonal treatment taken. **b**, Overview of the number of donors and cells per hormonal treatment taken in each dataset profiled by scRNA-seq. **c**, Bar plot showing the cellular composition of endometrial biopsies from donors taking the different hormonal treatment (y-axis) in the scRNA-seq data. **d**, UMAP projections of the snRNA-seq data coloured by hormonal treatment taken. **e**, Overview of the number of donors and cells per hormonal treatment taken profiled by snRNA-seq. **f**, Bar plot showing the cellular composition of endometrial biopsies from donors taking the different hormonal treatment (y-axis) in the snRNA-seq dataset. dStromal, decidualised stromal cells; ePV, endometrial perivascular cells; eStromal, endometrial stromal cells specific to proliferative phase; MMPs, matrix metalloproteinases; mPV, myometrial perivascular cells; Prolif., proliferative; scRNA-seq; single-cell RNA-sequencing; secret., secretory; snRNA-seq, single-nucleus RNA-sequencing; UMAP, uniform manifold approximation and projection; uSMCs, uterine smooth muscle cells.

As expected, most of the cell populations were of endometrial origin, but the atlas also contained populations exclusively present in the myometrium from the whole-uterine samples (for example, uterine smooth muscle cells (uSMCs) and myometrial PV cells). In addition, we detected a small number of mesenchymal HOXA13^+^ and epithelial KRT5^+^ cells, which based on their marker gene expression were likely cervical cell contamination. This was supported by their transcriptomic similarity to cervical cells when we compared the HECA with a publicly available scRNA-seq dataset of the cervix^30^ (Extended Data Fig. 1e–i). We did not detect any endometriosis-specific cell state in either the scRNA-seq or the snRNA-seq data, providing further evidence that at the cellular level of the endometrium, differences between controls and cases may be more subtle. However, additional cell states appeared in samples from donors taking exogenous hormones, indicating that exogenous hormones strongly impact the global transcriptome of epithelial cells, an observation supported by both data sources (Extended Data Fig. 5a–f).

Altogether, we generated the most comprehensive reference atlas of the human endometrium to date—the HECA. Researchers can map and contextualize newly processed samples onto the HECA following the computational tutorials in Supplementary Note 2.

### Spatiotemporal complexity of the endometrial epithelium

The endometrial epithelium consists of a complex network of basalis glands, functionalis glands extending into the uterine cavity and a layer of luminal cells (Fig. 1a). Here, we thoroughly characterized the cell states forming the different regions of the endometrial epithelium across the proliferative and secretory phases.

**Fig. 2:**
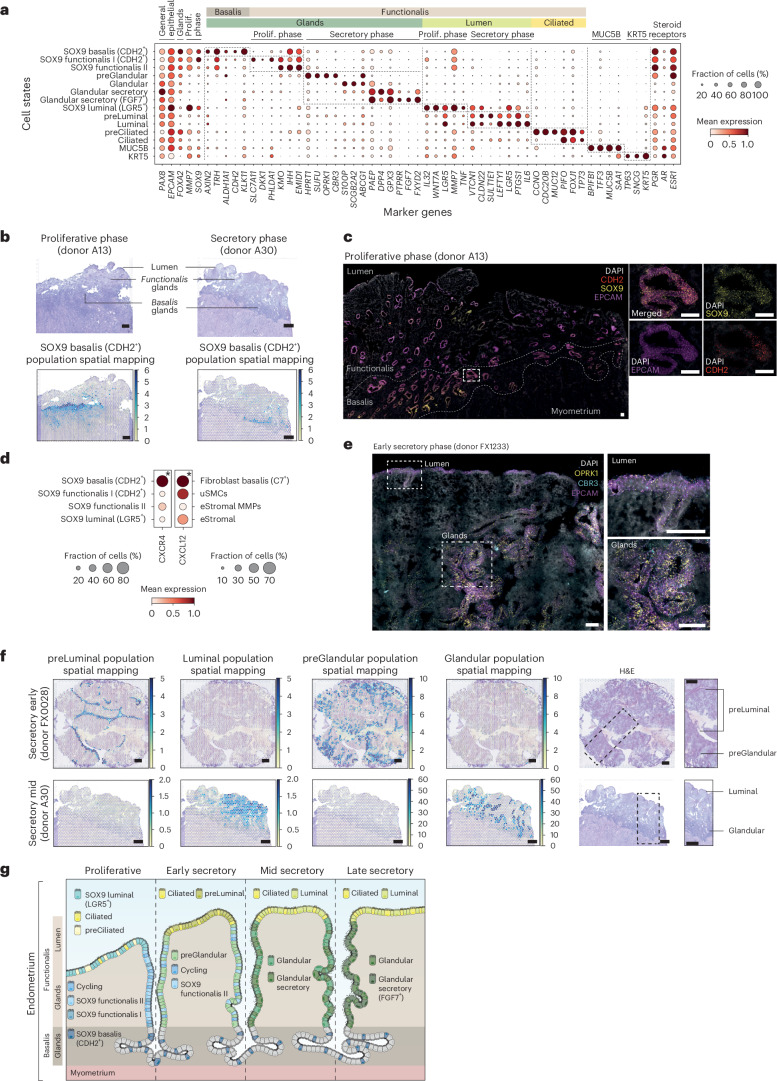
Spatiotemporal complexity of epithelial cells. **a**, Dot plot showing normalized, log-transformed and variance-scaled expression of genes (*x* axis) for epithelial cell states (*y* axis) in scRNA-seq data. **b**, Visium spatial transcriptomics data and an H&E image of the same tissue section. Spot color indicates cell2location-estimated cell density for the SOX9 basalis (CDH2^+^) population in sections of whole-uterus biopsies (*n* = 2 biologically independent samples) from donors A13 (proliferative phase) and A30 (secretory phase). **c**, High-resolution multiplexed smFISH of a section of a whole-uterus biopsy from donor A13 stained for DAPI (white, nuclei), EPCAM (magenta, epithelium), SOX9 (yellow, epithelium) and CDH2 (red, basalis epithelium) (*n* = 2 biologically independent samples). The dotted line highlights the basalis endometrium where signals for all markers co-localize within the basalis glands. The inset shows a representative magnified area. Scale bars, 100 µm. **d**, Dot plot showing normalized, log-transformed and variance-scaled expression of *CXCR4* and *CXCL12* (*x* axis) in a selection of epithelial and mesenchymal cells (*y* axis) in scRNA-seq data. Asterisk denotes a significant cell–cell interaction identified through CellPhoneDB analyses. **e**, Left, high-resolution multiplexed smFISH of a section of a superficial biopsy from donor FX1233 showing the expression of *DAPI* (white, nuclei), *EPCAM* (magenta, epithelium), *CBR3* (cyan, preGlandular cells) and *OPRK1* (yellow, preGlandular cells) (*n* = 2 biologically independent samples). Top right, a magnified image of the luminal region with low *OPRK1* and *CBR3* signal. Bottom right, a magnified image of the glandular region with high and co-localized *OPRK1* and *CBR3* signal. Scale bars, 100 µm. **f**, Visium spatial transcriptomics data and an H&E image of the same tissue section. Spot color indicates cell2location-estimated cell density for the preLuminal, Luminal, preGlandular and Glandular populations in a section of a superficial biopsy from donor FX0028 (early secretory phase; *n* = 2 biologically independent samples) and a section of a whole-uterus biopsy from donor A30 (mid secretory phase; *n* = 1). Scale bars, 1 mm. **g**, Schematic illustration of the spatiotemporal complexity of the endometrial epithelium across the proliferative and secretory phases.

We identified a population of SOX9^+^ basalis (*CDH2*^+^) cells that was not reported by previous single-cell transcriptomics atlases. These cells expressed markers described for endometrial epithelial stem/progenitor cells (*SOX9*, *CDH2*, *AXIN2*, *ALDH1A1* (refs. ^9,31–34^)) (Fig. 2a). Using spatial transcriptomics and single-molecule fluorescence in situ hybridization (smFISH) imaging, we mapped this population to the basalis glands region in full thickness endometrial biopsies from both proliferative and secretory phases (Fig. 2b,c).

**Extended Data Fig. 6:**
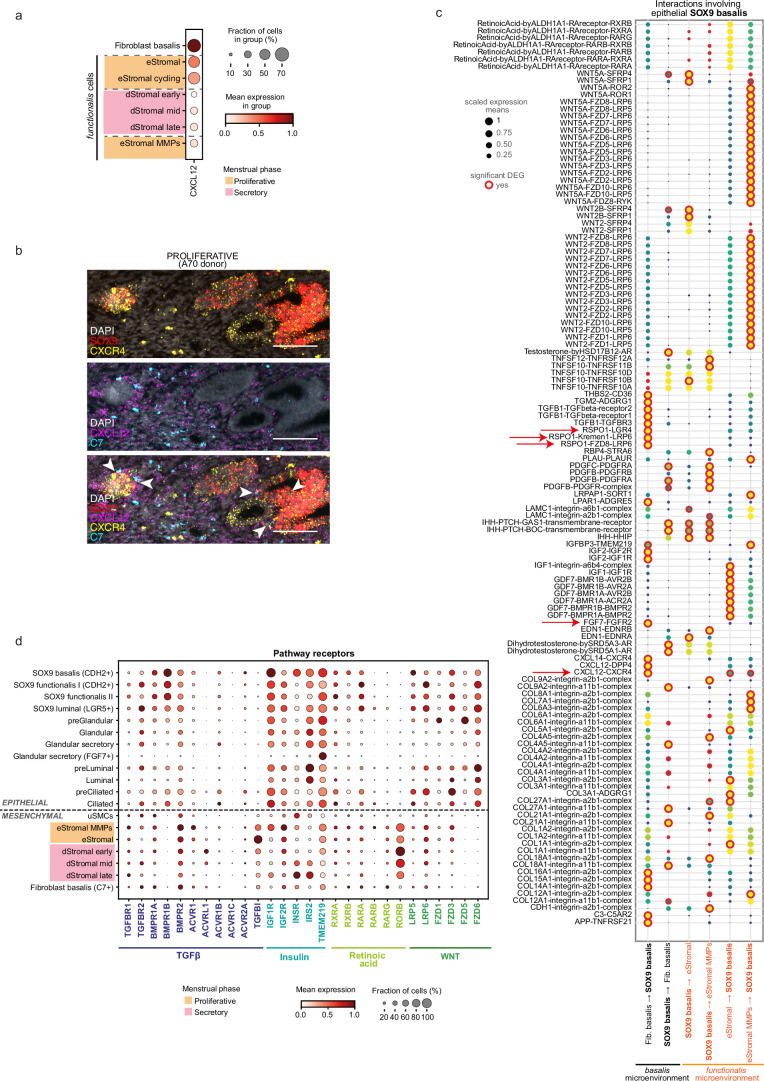
Expression of ligands and receptors involved in epithelial- stromal cell communication. **a**, Dotplot plot reporting the variance-scaled mean expression of CXCL12 (ligand of CXCR4). Red circles indicate that at least one of the interacting partners is differentially expressed in one of the cell types in the pair. **b**, High-resolution multiplexed smFISH of endometrium section (donor A70; n = 2 biologically independent samples) showing the expression of DAPI (white), SOX9 (red), CXCR4 (yellow), CXCL12 (magenta), C7 (cyan). White arrows indicate regions where all signals can be detected in high proximity. Scale bars = 100 µm. **c**, Dotplot plot reporting the variance-scaled mean expression of the two or more (if heteromeric complexes) transcripts coding for the interacting proteins in pairs of cell types. Red circles indicate that at least one of the interacting partners is differentially expressed in one of the cell types in the pair. **d**, Dot plot showing normalised, log-transformed and variance-scaled expression of genes coding for TGFβ, insulin, retinoic acid and WNT signalling receptors (x-axis) in the epithelial and mesenchymal cell states identified (y-axis) in the scRNA-seq data. eStromal, endometrial stromal cells specific to proliferative phase; dStromal, decidualised stromal cells; MMPs, matrix metalloproteinases; scRNA-seq, single-cell RNA-sequencing; TGFβ, transforming growth factor beta; uSMCs, uterine smooth muscle cells; smFISH, single molecule fluorescence in situ hybridisation.

Cell–cell interaction analyses indicated that the SOX9 basalis (*CDH2*^+^) population interacts with the fibroblast basalis (that is, fibroblast basalis *C7*^+^) population via the expression of *CXCR4* and *CXCL12*, respectively (Fig. 2d and Extended Data Fig. 6a,b). In addition, we detected an enrichment of interactions that suggest active WNT (*RSPO1/LGR4/LRP6*) and fibroblast growth factor (FGF; *FGF7/FGFR2*) signaling (Extended Data Fig. 6c, Supplementary Note 3 and Supplementary Table 4). CXCL12, WNT and FGF signaling are known to have a role in the maintenance of the stem cell niche in other tissues^35–41^, suggesting the existence of a signaling center that favors the maintenance of the endometrial SOX9 basalis (*CDH2*^+^) population, in a manner typical of a stem cell niche.

**Extended Data Fig. 7:**
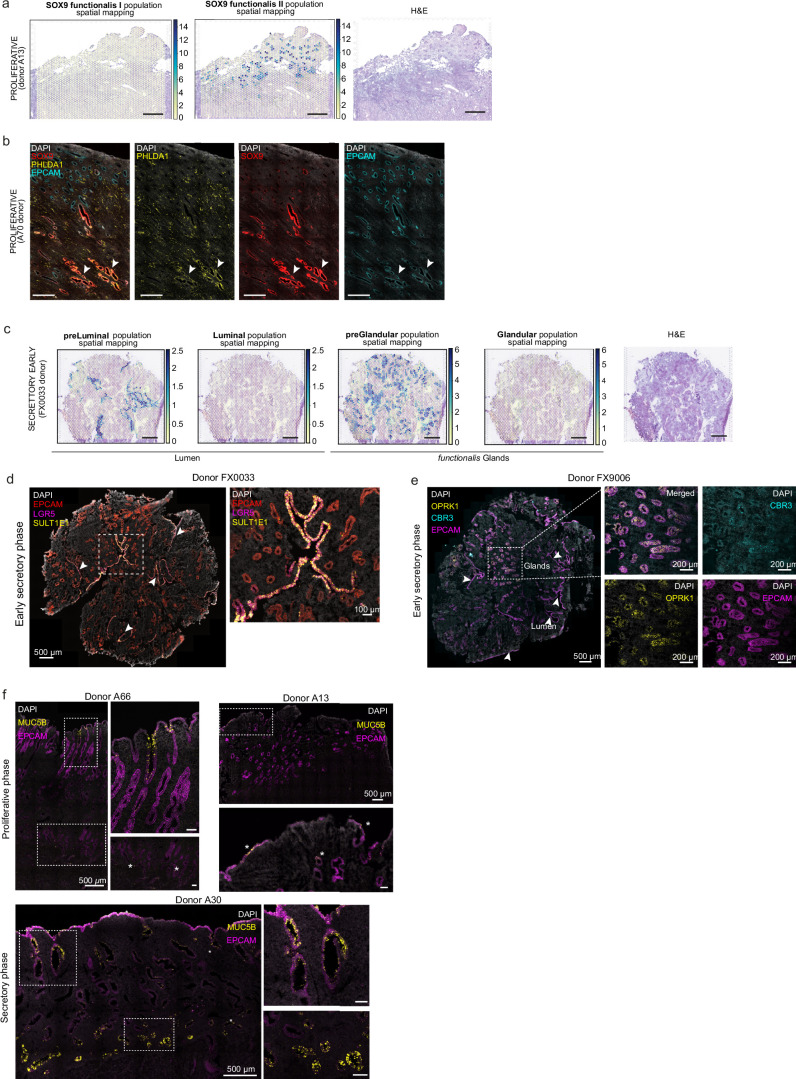
Spatial mapping of epithelial cell populations. **a**, Visium spatial transcriptomics data and an H&E image of a full thickness uterine section (donor A13, n = 2 independent samples from the same donor). Spot colour indicates cell2location-estimated cell density for SOX9 functionalis I and II populations. Scale bars = 1 mm. **b**, High-resolution multiplexed smFISH of full thickness endometrium sections (donor A70; n = 3 biologically independent samples) showing the expression of DAPI (white, nuclei), PHLDA1 (yellow, SOX9 Functionalis I cells), SOX9 (red, SOX9+ epithelium), and EPCAM (cyan, epithelium). White arrows indicate PHLDA1-expressing SOX9 Functionalis I cells. Scale bars = 500 µm. **c**, Visium spatial transcriptomics data and an H&E image of the same superficial biopsy section (donor FX0033, early secretory phase; n = 2 biologically independent samples). Spot colour indicates cell2location-estimated cell density for the preLuminal, Luminal, preGlandular and Glandular populations. Scale bars = 1 mm. **d**, High-resolution multiplexed smFISH of a superficial biopsy section (donor FX0033, early secretory phase; n = 3 biologically independent samples) showing the expression of DAPI (white, nuclei), EPCAM (red, epithelium), LGR5 (magenta, luminal cell), and SULT1E1 (yellow, preLuminal cells). White arrows indicate luminal regions with high LGR5 and SULT1E1 signals. The dashed outline indicates the magnified area of the luminal region with high and co-localised LGR5 and SULT1E1 signals. **e**, High-resolution multiplexed smFISH of a superficial biopsy section (donor FX9006, early secretory phase; n = 2 biologically independent samples) showing the expression of DAPI (white, nuclei), EPCAM (magenta, epithelium), CBR3 (cyan, preGlandular cells), and OPRK1 (yellow, preGlandular cells). The dashed outline indicates a magnified area of the glands with high and co-localised OPRK1 and CBR3 signals. White arrows indicate luminal regions with low OPRK1 and CBR3 signals. **f**, High-resolution multiplexed smFISH of full thickness endometrium sections from the proliferative phase (donors A66 and A13) and secretory phase (donor A30) showing the expression of DAPI (white, nuclei), EPCAM (magenta, epithelium), and MUC5B (yellow, MUC5B cells) (n = 3 biologically independent samples). The dashed outline indicates the magnified areas. Asterisks indicate representative regions where the MUC5B signal was detected and varied across samples. Scale bars = 100 µm, unless differently specified. smFISH, single molecule fluorescence in situ hybridisation.

The cellular composition of the functionalis glands showed highly dynamic changes across the proliferative and secretory phases (Fig. 2a). During the proliferative phase, we uncovered further heterogeneity within the known SOX9^+^ cell population^15^. Specifically, we identified two SOX9^+^ cell states: SOX9 functionalis I and II, which we mapped to the functionalis glands (Extended Data Fig. 7a). The SOX9 functionalis I population expressed *CDH2* and high levels of *SOX9* and was marked by the expression of *PHLDA1* and *SLC7A11* (Extended Data Fig. 7b). It also expressed the WNT inhibitor *DKK1*, and, in line with this, *AXIN2* was downregulated in both SOX9 functionalis states (Fig. 2a). The SOX9 functionalis II population exhibited lower expression of *SOX9* and *CDH2*, and distinctly expressed *KMO*, *IHH* and *EMID1*. The luminal proliferative epithelium was defined by the presence of SOX9 luminal (*LGR5*^+^), pre-ciliated and ciliated cells (Figs. 1f and 2a), as we described previously^15^. As expected, a larger proportion of cycling epithelial cells was detected in the proliferative phase (Fig. 1f).

During the secretory phase, the SOX9^+^ populations were markedly reduced as the endometrium underwent further differentiation to prepare a receptive environment for blastocyst implantation (Fig. 1f). Having a larger number of samples allowed us to further subdivide the secretory phase into early, early-mid, mid and late secretory phases and to define the populations associated with these stages (Fig. 1f). We uncovered the transcriptomic profiles of cells characteristic of the functionalis layer during the early secretory phase (that is, the preGlandular and preLuminal populations; Fig. 2a,e,f). These populations were transcriptomically similar to the previously described glandular and luminal populations^15^, but appeared at earlier stages of the secretory phase (after the progesterone surge) and expressed markers not defined previously. *OPRK1*, *SUFU*, *CBR3* and *HPRT1* were specific to the preGlandular population and *SULT1E1* to the preLuminal population (Fig. 2a). Using spatial transcriptomics, we confidently mapped both populations to early but not mid secretory samples. Specifically, the preLuminal population mapped to the lumen and the preGlandular population to the functionalis glands (Fig. 2f and Extended Data Fig. 7c), which we further confirmed by smFISH (Fig. 2e and Extended Data Fig. 7d,e).

The number of preGlandular and preLuminal cells decreased in the early-mid and mid secretory phase samples, with the dominant cell states being the previously described glandular, luminal and ciliated populations^15^ (Fig. 1f). Lastly, analyzing a single sample from the late secretory phase, we observed the presence of a glandular secretory population that upregulated *FGF7*, a mitogen with a wound healing role in other contexts^42,43^.

We detected a previously described population of MUC5B^+^ epithelial cells^16^ expressing *MUC5B*, *TFF3*, *SAA1* and *BPIFB1*. As in previous studies^16^, we also observed varied expression of the cell type marker *MUC5B* when staining full thickness endometrial biopsies using smFISH (Extended Data Fig. 7f). However, when projecting a publicly available scRNA-seq dataset of the cervix^30^ onto our HECA (Extended Data Fig. 1h), we found a cluster of cervical epithelial cells matching the transcriptome of this population (Extended Data Fig. 1g–i). This result implies the MUC5B cells are likely to be present in the endocervical columnar epithelial cells^30,44^, and we cannot disregard the possibility that in the HECA, the MUC5B population comes exclusively from the endocervix.

In summary, we defined and spatially located previously unreported epithelial cell states across the proliferative and secretory phases, including a putative stem/progenitor cell population found within the basalis and multiple transitory cell states dominating the functionalis (Fig. 2g).

### Stromal–epithelial cell crosstalk across the menstrual cycle

During the menstrual cycle, stromal and epithelial cells synchronize their differentiation under the influence of ovarian hormones, as well as locally produced paracrine factors. Here we used the HECA’s fine-grained classification of stromal and epithelial cell states across the menstrual cycle to infer cell–cell communication occurring in vivo along the endometrial niches in space (that is, basalis, functionalis) and time (that is, menstrual cycle phases).

**Fig. 3:**
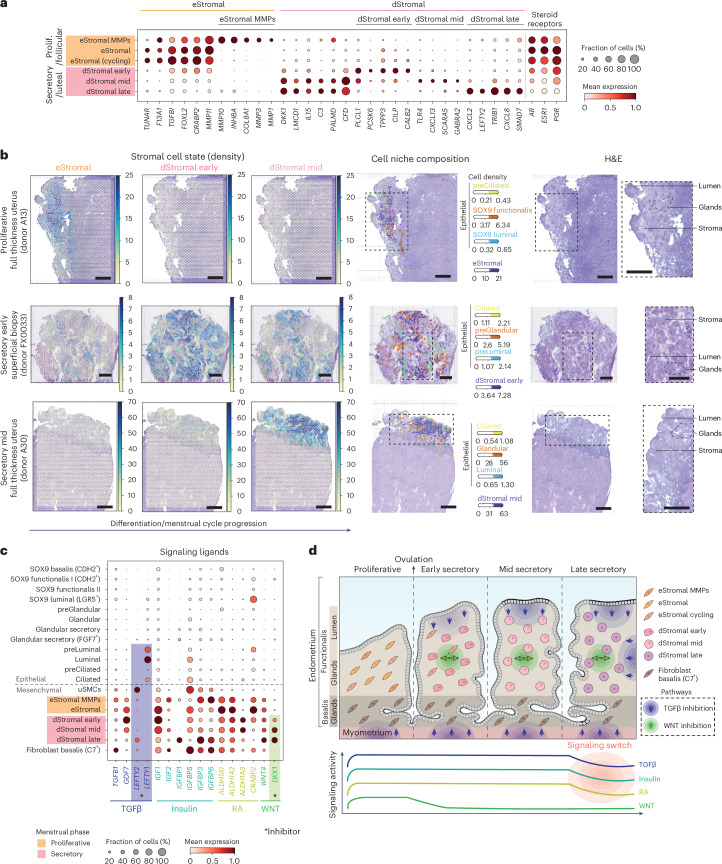
Endometrial stromal cell heterogeneity and stromal–epithelial cell crosstalk across the menstrual cycle. **a**, Dot plot showing normalized, log-transformed and variance-scaled expression of genes (*x* axis) characteristic of the identified stromal cell states (*y* axis) in scRNA-seq data. **b**, Visium spatial transcriptomics data and an H&E image of the same tissue section are shown. Spot color indicates estimated cell state density for a specific cell population in each Visium spot as computed by cell2location. Spatial mapping of the eStromal, dStromal early and dStromal mid cell populations is shown in a section of a whole-uterus biopsy from donor A13 (top panel, proliferative phase; a representative image of *n* = 2 independent samples from the same donor), a section of a superficial biopsy from donor FX0033 (middle panel, early secretory phase; a representative image of *n* = 2 biologically independent samples) and a section of a whole-uterus biopsy from donor A30 (bottom panel, mid secretory phase; a representative image of *n* = 2 independent samples from the same donor). Mapping of menstrual cycle phase-relevant epithelial cell populations is also shown in the niche composition panel. Scale bars, 1 mm. **c**, Dot plot showing normalized, log-transformed and variance-scaled expression of genes coding for ligands involved in TGFβ, insulin, retinoic acid and WNT signaling (*x* axis) in epithelial and mesenchymal cell states (*y* axis) in scRNA-seq data. **d**, Schematic illustration of the temporal complexity of endometrial stromal cells and signaling pathways across the proliferative and secretory phases. RA, retinoic acid.

Within the functionalis layer, endometrial stromal cells specific to the proliferative phase (eStromal cells) and decidualized stromal cells specific to the secretory phase (dStromal cells) were defined previously at the single-cell level^15^. In the HECA, we further identified a type of eStromal cells (eStromal matrix metalloproteinases (MMPs)) in samples collected during the menstrual and early proliferative phases (Fig. 3a, Extended Data Fig. 1d and Supplementary Note 4), characterized by the upregulation of metalloproteases (*MMP1*, *MMP10*, *MMP3*) and inhibin A (*INHBA*) (Fig. 3a).

In secretory phase samples, we uncovered three dStromal cell states appearing at different stages of the secretory phase. Early decidualized stromal cells (dStromal early) were enriched in the early secretory phase samples and upregulated the progesterone-induced gene *PLCL1* (ref. ^45^) (Fig. 3a,b). The mid decidualized stromal population (dStromal mid) mapped to early-mid and mid secretory phase samples and upregulated *DKK1* (Fig. 3a,b), a WNT inhibitor crucial for the differentiation of epithelial secretory glands^15^. Late decidualized stromal cells (dStromal late) were present in both mid and late secretory phase samples (Fig. 1f) and upregulated the premenstrual marker *LEFTY2* (ref. ^46^) (Fig. 3a). Both the dStromal mid and late populations downregulated estrogen and progesterone receptors (*ESR1* and *PGR*).

We uncovered a putative intricate spatiotemporal regulation of transforming growth factor beta (TGFβ) signaling (Fig. 3c). Specifically, the TGFβ superfamily receptors were ubiquitously expressed by all epithelial and stromal cells at all stages of the menstrual cycle (Extended Data Fig. 6d). Meanwhile, the ligands of TGFβ and growth differentiation factor (GDF) subfamilies (*TGFB1* and *GDF7*, respectively) were upregulated by all stromal cells until mid/late secretory phase, when expression dropped (Fig. 3c). Interestingly, the activity of TGFβ signaling appeared confined within specific spatial and temporal boundaries by its antagonists, *LEFTY1* and *LEFTY2*. On one hand, *LEFTY1* was expressed by epithelial cells of the lumen (ciliated and luminal) and *LEFTY2* by uSMCs of the myometrium (Fig. 3c), establishing a top–bottom spatial boundary of TGFβ activity. On the other hand, the temporal boundary seemed to be determined by the expression of *LEFTY2* as well as *SMAD7* (the inhibitor of SMAD proteins, downstream effectors of TGFβ), expressed by the dStromal late population (Fig. 3a) towards the end of the menstrual cycle (Fig. 3c).

Taken together, our data supported a potential rise in TGFβ, WNT^47^, insulin^48^ and retinoic acid^49^ signaling from early stages of the proliferative phase (Fig. 3d). WNT inhibition (via *DDK1*) marked the beginning of the secretory phase with the decidualization of stromal cells. In the late secretory phase, our data supported a signaling switch in the use of TGFβ signaling, insulin growth factors and retinoic acid metabolism (Fig. 3c,d). The full collection of cell–cell communication factors, identified through CellPhoneDB analyses^50^, can be visualized in our CellPhoneDBViz portal (https://www.cellphonedb.org/viz/viz.html?projectid=harmonized_endometrial_atlas&auth=7xWkX47Qatox6dikwb-TgA).

### Macrophages in endometrial regeneration

**Fig. 4:**
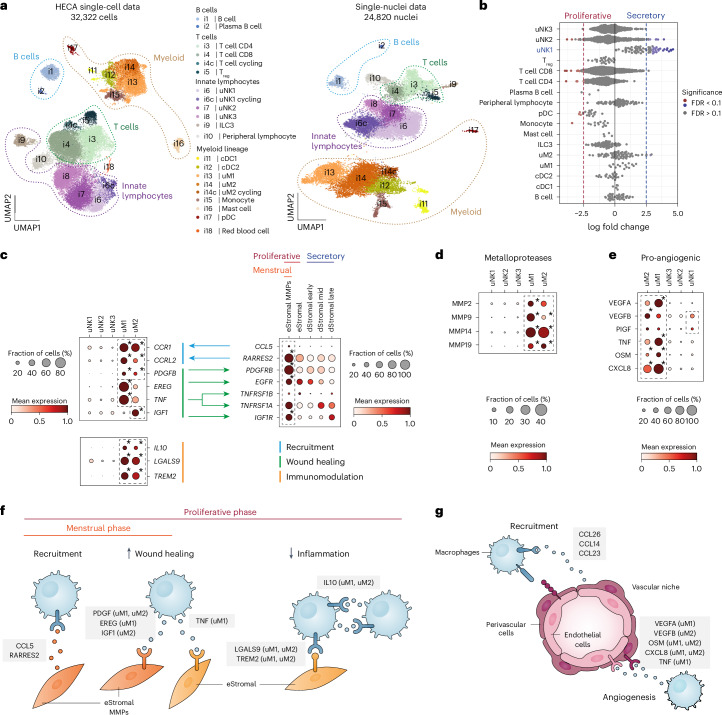
Predicted ligand–receptor interactions and role of macrophages in endometrial repair and regeneration. **a**, Left, UMAP projections of scRNA-seq data for 32,322 immune cells colored by cell type. Right, UMAP projections of snRNA-seq data for 24,820 immune cells/nuclei colored by cell type. **b**, Beeswarm plot of the distribution of log fold change between the proliferative and secretory phases in neighborhoods containing immune cells from different cell types in scRNA-seq data. Differentially abundant neighborhoods at log fold change > 2.5 and spatial FDR < 0.1 are colored. **c**, Dot plot showing normalized, log-transformed and variance-scaled expression of genes (*y* axis) in uNK and uM cell states (*x* axis) in scRNA-seq data. Asterisk denotes significantly upregulated expression at FDR < 0.05. **d**, Dot plots showing normalized, log-transformed and variance-scaled expression of signaling molecules and receptors (*y* axes) upregulated in uNK, uM and stromal cell states (*x* axes) in scRNA-seq data. Asterisk denotes significantly upregulated expression at FDR < 0.05. The predicted cell–cell communication between uNK, uM and stromal cell states, including its likely role, is shown by differently colored arrows. **e**, Dot plot showing normalized, log-transformed and variance-scaled expression of pro-angiogenic signaling molecules (*y* axis) upregulated in uNK and uM cell states (*x* axis) in scRNA-seq data. Asterisk denotes significantly upregulated expression at FDR < 0.05. **f**, Schematic illustration of macrophage and stromal cell signaling during the menstrual and proliferative phases, likely involved in macrophage cell recruitment, increasing wound healing abilities and dampening inflammation in stromal cells. **g**, Schematic illustration of macrophage, endothelial cell and PV cell signaling likely involved in macrophage recruitment and angiogenesis. Cells from donors on hormones and donors with endometriosis were excluded from analyses shown in **b**–**e** of this figure. Asterisk denotes significantly upregulated expression FDR < 0.05. cDC, conventional dendritic cells; FDR, false discovery rate; ILC3, innate lymphoid cell type 3; pDC, plasmacytoid dendritic cell; T_reg_, regulatory T cells.

**Extended Data Fig. 8:**
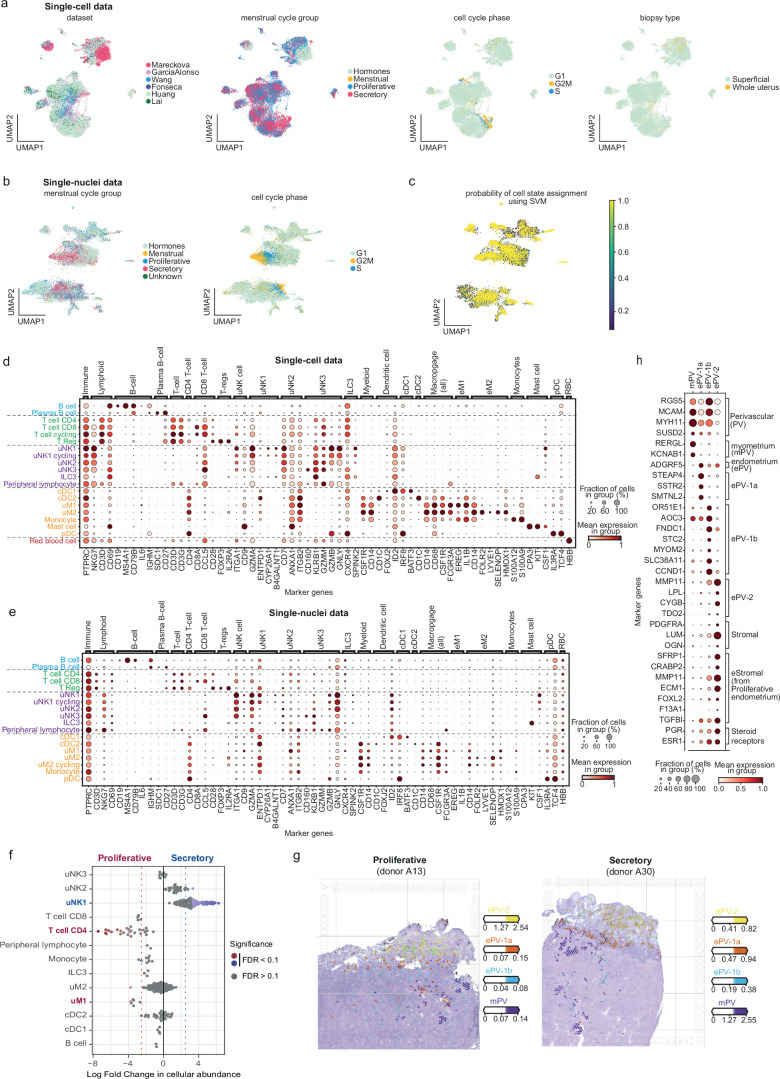
Immune cells in scRNA-seq and snRNA-seq data. **a**, UMAP projections of scRNA-seq data for immune cells coloured by dataset, menstrual cycle group, cell cycle phase and biopsy type. **b**, UMAP projections of snRNA-seq data for immune cells coloured by menstrual cycle group and cell cycle phase. **c**, UMAP projection of snRNA-seq data for immune cells coloured by the probability of assigning the immune cell types identified in the scRNA-seq data. Support Vector Machine (SVM) classifier was trained using the immune cell scRNA-seq data and the predicted cell type annotations were then projected onto the snRNA-seq data with the probability shown. **d**, Dot plot showing normalised, log-transformed and variance-scaled expression of genes (x-axis) characteristic of the identified immune cell states (y-axis) in the scRNA-seq data. **e**, Dot plot showing normalised, log-transformed and variance-scaled expression of genes (x-axis) characteristic of the identified immune cell states (y-axis) in the snRNA-seq data. **f**, Beeswarm plot of the distribution of log fold change across the menstrual cycle (proliferative and secretory phases) in neighbourhoods containing immune cells from different cell type clusters in snRNA-seq data. Differentially abundant neighbourhoods at log fold change > 2.5 and spatial FDR < 0.1 are coloured. **g**, Visium spatial transcriptomics data for donors A13 (proliferative phase) and A30 (secretory phase) (n = 2 biologically independent samples) are shown. Spot colour indicates estimated cell state density for a specific population of perivascular cells (mPV, ePV-1a, ePV-1b and ePV-2) in each Visium spot, as computed by cell2location. **h**, Dot plot showing normalised, log-transformed and variance-scaled expression of genes (x-axis) characteristic of the identified endothelial, perivascular and stromal cells (y-axis) in the scRNA-seq data. cDC, conventional dendritic cells; eStromal, endometrial stromal cells specific to proliferative phase; ePV, endometrial perivascular cells; FDR, false discovery rate; ILC3, innate lymphoid cell type 3; mPV, myometrial perivascular cells; pDC, plasmacytoid dendritic cells; RBC, red blood cells; scRNA-seq, single-cell RNA-sequencing; snRNA-seq, single-nucleus RNA-sequencing; SVM, support vector machine; T Reg, T regulatory cells; uM, uterine macrophages; UMAP, uniform manifold approximation and projection; uNK, uterine natural killer cells.

To gain insights into the diversity and dynamics of innate immune cells in the regeneration and differentiation of the endometrium in natural menstrual cycles, we annotated the immune compartment (*n* = 32,322 cells and *n* = 24,820 nuclei; Methods). These datasets captured the three uterine natural killer cell (uNK) populations (uNK1, uNK2, uNK3) and the two uterine macrophage (uM) populations (uM1 and uM2) previously identified by us in the endometrium during pregnancy (that is, decidua)^51^ (Fig. 4a and Extended Data Fig. 8a–e). uM1 expressed pro-inflammatory genes such as *IL1B* and *EREG*, while uM2 expressed anti-inflammatory genes such as *HMOX1* (ref. ^52^). uM2 also expressed tissue-resident macrophage markers such as *FOLR2* and *LYVE1* (ref. ^53^) (Extended Data Fig. 8d,e). Differential cell abundance analysis (Supplementary Note 5) demonstrated an increase in the abundance of uNK1 cells during the secretory phase, in line with previous reports of granular endometrial immune cells proliferating during the secretory phase^54,55^ (Fig. 4b and Extended Data Fig. 8f). We did not detect cell abundance changes of the other immune cell types between the proliferative and secretory phases.

**Extended Data Fig. 9:**
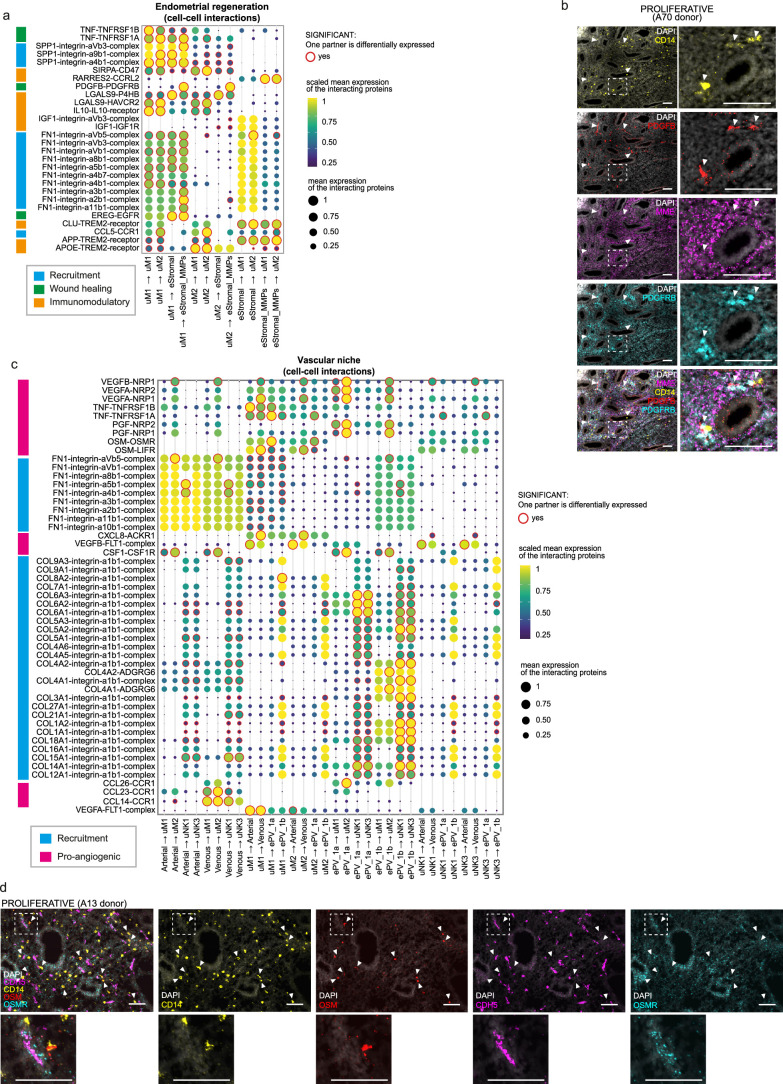
Predicted cell-cell interactions underpinning endometrial regeneration and angiogenesis. **a**, Dotplot plot reporting the variance-scaled mean expression of the two or more (if heteromeric complexes) transcripts coding for the interacting proteins in pairs of cell types. Red circles indicate that at least one of the interacting partners is differentially expressed in one of the cell types in the pair. Interactions are classified based on whether they are predicted to play a role in recruitment, wound healing or immunomodulation during endometrial regeneration. **b**, High-resolution multiplexed smFISH of full thickness endometrium sections (donor A70; n = 3; independent samples from the same donor) showing the expression of DAPI (white, nuclei), CD14 (yellow, macrophages), PDGFB (red), MME (magenta, eStromal MMPs), PDGFRB (cyan, PDGFB receptor). The dashed outline indicates the area shown magnified to the right. White arrows indicate regions of interaction between macrophages and eStromal MMPs by means of signal colocalization and/or proximity. Scale bars = 100 µm. **c**, Dotplot plot reporting the variance-scaled mean expression of the two or more (if heteromeric complexes) transcripts coding for the interacting proteins in pairs of cell types. Red circles indicate that at least one of the interacting partners is differentially expressed in one of the cell types in the pair. Interactions are classified based on whether they are predicted to play a role in cell recruitment or pro-angiogenic processes within the vascular niche. **d**, High-resolution multiplexed smFISH of full thickness endometrium sections (donor A13; n = 3; independent samples from the same donor) showing the expression of DAPI (white, nuclei), CD14 (yellow, macrophages), OSM (red), CDH5 (magenta, endothelial cells), OSMR (cyan, OSM receptor). The dashed outline indicates the area shown magnified underneath. White arrows indicate regions of interaction between macrophages and endothelial cells by means of signal colocalization or proximity. Scale bars = 100 µm. smFISH, single molecule fluorescence in situ hybridisation.

To deepen our understanding of the roles uMs and uNKs play in endometrial regeneration, we interrogated their cell–cell communication with stromal, endothelial and PV cells. We found that the eStromal MMPs population (characteristic of the menstrual phase) expressed integrins and cytokines (*CCL5*, *RARRES2*) which can bind their cognate receptors upregulated by uMs (*CCR1*, *CCRL2*) (Fig. 4c, Extended Data Fig. 9a and Supplementary Table 4). This interaction likely supports the previously described recruitment of uMs to the tissue during menstruation^56,57^. Both uM1 and uM2 upregulated *PDGFB*, a protein from the PDGF family, known for its role in wound healing and repair in various tissues^58,59^. In the endometrium, it could operate by binding to the *PDGFRB* receptor, which is upregulated by eStromal MMPs and also present in the other stromal cells (Fig. 4c and Extended Data Fig. 9b). Additionally, uMs upregulated *TNF* (uM1), as well as growth factors such as *IGF1* (uM2) and *EREG* (uM1). These could stimulate the proliferation and survival of eStromal MMPs and proliferative eStromal cells by binding to their corresponding receptors (*EGFR*, *TNFRSF1A*, *TNFRSF1B* and *IGF1R*) (Fig. 4c). Finally, both uMs also expressed immunoregulatory genes (*IL10*, *LGALS9*, *TREM2*) that could enhance anti-inflammatory responses in the proliferative phase endometrium required for the characteristic scarless regeneration of this tissue (Fig. 4c).

Angiogenesis is also critical for tissue repair, and macrophages are known to play a role in this process^60^. To investigate the potential interplay between uMs and endometrial vasculature, we first defined the vascular niche. We identified three subsets of endothelial cells (venous, arterial and lymphatic) and three subsets of endometrial PV cells (ePV-1a expressing *STEAP4*, ePV-1b expressing *STC2* and ePV-2 expressing *MMP11*) (Extended Data Fig. 8g,h). ePV-2 exhibited transcriptomic similarities to endometrial stromal cells, suggesting a transitional population between PV and stromal cells (Extended Data Fig. 1c).

Cell–cell communication analyses predicted signaling between the vasculature and uMs, and to a lesser extent also with uNK1 cells. Endothelial cells and ePV-1s expressed multiple extracellular matrix proteins and cytokines (*CCL14*, *CCL23*, *CCL26*), which potentially could act to recruit innate immune cells (Extended Data Fig. 9c and Supplementary Table 4). Additionally, PV cells expressed *CSF1* (major macrophage growth factor), which could create a favorable environment for macrophages, stimulating their differentiation and function. In turn, uMs expressed multiple growth factor members of the pro-angiogenic VEGF family (*VEGFA* in uM1 and *VEGFB* in uM2) and vascular remodeling factors (*TNF*^61^ in uM1 and *OSM*^62^ and *CXCL8* (ref. ^63^) in both uMs), whose cognate receptors (*NRP1*, *NRP2*, *FLT1*, *TNFRSF1A-B*, *OSMR*, *LIFR*, *ACKR1*) were expressed by the endothelial cells (Fig. 4e and Extended Data Fig. 9c,d). Among the innate lymphocytes, uNK1 was the only cell subset that expressed pro-angiogenic factors (*VEGFB* and *PIGF*), although at lower levels than uMs (Fig. 4e).

Altogether, our analyses suggested that uMs are the major endometrial immune cells involved in blood vessel formation, wound healing and anti-inflammatory responses (Fig. 4f,g). The latter two processes are likely to aid the stromal cells in healing without scarring.

### Altered stromal–immune cell homeostasis in endometriosis

**Fig. 5:**
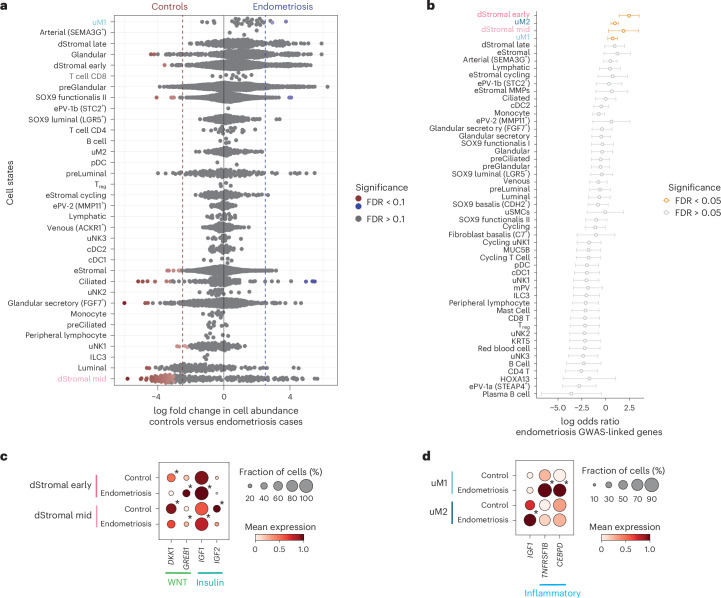
Endometrial stromal–immune cell niche in endometriosis. **a**, Beeswarm plot of cellular composition changes between controls and endometriosis cases detected by RMilo’s differential cell abundance test in the snRNA-seq dataset. Donors taking exogenous hormones were excluded from the analysis. Each dot represents the log fold change between conditions (that is, controls versus endometriosis cases) of a cell type neighborhood. Cell neighborhoods at log fold change > 2.5 and spatial FDR < 0.1 are colored. **b**, Forest plot of each endometrial cell type (*y* axis) representing the enrichment for expression of genes (log odds ratio (*x* axis)) associated with endometriosis, estimated from the fGWAS test. Data are presented as log odds ratio ± 95% CI. Cell types in orange have FDR < 0.05. **c**, Dot plot showing normalized, log-transformed and variance-scaled expression of differentially expressed genes (*x* axis) in dStromal cell states of controls and endometriosis cases (*y* axis) in the scRNA-seq data. **d**, Dot plot showing normalized, log-transformed and variance-scaled expression of differentially expressed genes (*x* axis) upregulated in uM cell states (*y* axis) in the scRNA-seq data. Cells from donors on hormones were excluded from all analyses shown in this figure. Asterisks denote differentially expressed genes between controls and cases at FDR < 0.1. 95% CI, confidence interval.

We next investigated whether cellular composition of the endometrium differs between endometriosis cases and controls during natural menstrual cycles, as we did not detect any endometriosis-specific cell types (Supplementary Note 5). After accounting for menstrual cycle phase, differential abundance analysis of our nuclei dataset revealed lower abundance of decidualized stromal cells (dStromal mid) and higher abundance of uM1 macrophages in endometriosis cases (Fig. 5a). Interestingly, decidualized stromal cells (dStromal early and dStromal mid) and macrophages (uM1 and uM2) were also identified as the top cell types enriched for the expression of genes positionally close to endometriosis risk variants when performing functional GWAS (fGWAS) analysis across the HECA cell types (Fig. 5b and Supplementary Note 6). The fGWAS analysis provided cellular context to a large-scale endometriosis GWAS meta-analysis^29^.

To further explore the four cell populations identified as endometriosis-relevant, we performed differential gene expression analyses between controls and endometriosis cases (Supplementary Tables 5 and 6 and Supplementary Note 7). In the stromal compartment of endometriosis cases, we observed changes in gene expression that are likely to alter the WNT and insulin signaling pathways (Fig. 5c). Specifically, *GREB1* (a GWAS-linked gene induced by WNT signaling^64,65^) was significantly upregulated while *DKK1* (WNT inhibitor) was significantly downregulated in both dStromal early and dStromal mid cells in endometriosis. These changes suggested sustained WNT signaling in the secretory phase endometrium of donors with endometriosis. Similarly, we observed a dysregulation of insulin growth factors *IGF1* (a GWAS-linked gene) and *IGF2*. In dStromal early and dStromal mid populations*, IGF1* was significantly upregulated while *IGF2* was significantly downregulated in endometriosis cases. *IGF1* and *IGF2* play roles in cell proliferation and differentiation^66,67^, suggesting dysregulation of these processes may occur in endometriosis. In the macrophage compartment, and in line with previous reports in mice^68^, we observed a significant upregulation of *IGF1* in uM2 of endometriosis cases (Fig. 5d). In the uM1 population, a significant increase in expression of inflammatory genes (*TNFRSF1B*, *CEBPD*) was detected in endometriosis, in keeping with previous reports of increased inflammation in endometriosis^69,70^.

Taken together, the identified shifts in cell abundance, disease-relevant populations through fGWAS and differential gene expression analyses suggest dysregulation of stromal–immune cell homeostasis in the endometrium of women with endometriosis.

## Discussion

Globally, millions of women are affected by endometrial/uterine disorders^22–24,71^, yet the endometrium and the role of its cellular heterogeneity in these pathologies have been hugely understudied compared with other human tissues and diseases^72^. In this study, we present the HECA, a comprehensive cellular atlas of the human endometrium assembled for individuals with/without endometriosis to date. The HECA provides a crucial step towards improving our understanding of endometrial cell heterogeneity in health and disease as it: (1) incorporates the largest number of cells and individuals; (2) presents consensus cell annotation across studies; (3) provides the most granular cell state annotation and cell spatial location in situ; (4) offers a platform for easy and rapid annotation of future scRNA-seq studies of the endometrium; and (5) enables the contextualization of genetic association screens for endometrial/uterine disorders.

By comprehensively analyzing and spatially mapping ~614,000 high-quality cells and nuclei from 121 individuals, we substantially surpassed the number of donors and cells profiled by the initial, pioneering endometrial single-cell studies^14–21^. The large sample size enabled us to identify previously unreported cell states at the single-cell level, including a population of *CDH2*^+^ (that is, N-cadherin) epithelial cells. This population’s marker gene expression^9,31–34^, localization within the basalis glands and predicted cell–cell communication with a basalis fibroblast population indicated that these cells could be the previously described epithelial stem/progenitor cells. Defining the transcriptomic profile of these cells opens new avenues for exploring their role in endometrial repair and regeneration, as well as disease pathophysiology. Additionally, we captured multiple previously unreported transitory cell states (for example, preLuminal, preGlandular, subsets of decidualized stromal cells) during the early/mid secretory phase—a dynamic period crucial for endometrial receptivity preparation in response to rising progesterone levels. A tightly regulated cellular response to the changing levels of ovarian hormones is essential for menstrual cycle progression, maintenance of tissue homeostasis and fertility. Thus, the identified cell states could present promising targets for therapy in endometrial/uterine disorders that are characterized by the disruption of hormone-dependent downstream signaling and cellular responses^73^.

Aside from ovarian hormones, locally produced paracrine factors are essential for menstrual cycle progression. We provided a detailed map (and an interactive platform) of the predicted in vivo cell–cell communication across the cycle, which is an important addition to the body of existing knowledge predominantly derived from in vitro cell cultures^74–76^. Of particular interest is how TGFβ activity is controlled by various epithelial and mesenchymal cell states in both space and time. The identification and detailed description of in vivo signaling pathways involved in menstrual cycle progression could now be used to refine the media used for culturing endometrial organoids, currently supplemented with TGFβ inhibitors^77,78^. Incorporating the spatial and temporal TGFβ signaling could improve the physiological response and differentiation of these cells when treated with hormones mimicking the menstrual cycle, and thus reduce some of the previously observed differences between in vivo and in vitro endometrial cells^15^. We also revealed a range of interactions by which uMs may aid the process of scarless endometrial regeneration, supporting previous research proposing a role for uMs in this process^79–81^. Interactions between uMs and stromal cells were most evident around menstruation, emphasizing the crucial role of uMs during this phase^82^. To further dissect the dialog between macrophages and stromal cells during endometrial repair and regeneration, additional samples from the menstrual phase should be analyzed. Understanding whether disruption of these macrophage–stromal interactions contributes to widely common menstrual disorders (for example, abnormal uterine bleeding) could pave new paths for the development of immunology-based treatment.

Lastly, we demonstrated the utility of the HECA to give cellular context to a large-scale endometriosis GWAS meta-analysis^29^. We identified two subtypes of decidualized stromal cells and macrophages as endometriosis-relevant. The observed dysregulation of stromal–immune cell homeostasis is in line with previous reports^16,20,27,28,83,84^, but, overall, findings have been inconsistent. Our current findings suggest a role of uM1 and uM2 macrophage populations in contributing to an abnormal inflammatory environment within the endometrium of patients with endometriosis. At the messenger RNA level, our data indicated sustained WNT and dysregulated insulin signaling to be a feature of the dStromal early/mid populations in endometriosis cases. This is in line with previous observations of downregulation of *IGF2* and impaired WNT inhibition in the endometrium of women with endometriosis during the secretory phase^85–87^. We previously showed that inhibition of WNT signaling by stromal cells in response to progesterone is crucial in supporting the differentiation of glandular epithelium^15^. Our current findings suggest that this process may be altered in endometriosis. Yet, the observed differences in expression were subtle (that is, individual genes exhibited small fold changes likely due to their combinatorial contribution), requiring further validation. The involvement of WNT and insulin pathways in progesterone-mediated cellular responses could now be tested using three-dimensional in vitro models of the endometrium encompassing both stromal and epithelial cells^88^.

In summary, the HECA is a large-scale integrated reference atlas of the human endometrium, providing a conceptual framework upon which future studies can be built. With all resources publicly available in an easy-to-access interactive format, the HECA offers a platform/tool for advancing research into endometrial physiology and disorders, as well as guiding the development of physiologically relevant in vitro model systems of the human endometrium.

## Methods

### Patient samples

Superficial endometrial samples collected for the Mareckova et al. dataset came from four studies: (1) Endometriosis Oxford (ENDOX), (2) Fibroids and Endometriosis Oxford (FENOX), (3) the Sanger Human Cell Atlasing Project and (4) the Immunology and Subfertility study. Both ENDOX (REC: 09/H0604/58) and FENOX (REC: 17/SC/0664) obtained ethical approval from the Central University Research Ethics Committee, University of Oxford. Yorkshire & The Humber–Leeds East Research Ethics Committee approved the Sanger Human Cell Atlasing Project (REC: 19/YH/0441). The Immunology of Subfertility study (REC: 08/H0606/94) was approved by the Oxford Research Ethics Committee C. In all instances, written, informed consent was provided by study participants before obtaining tissue samples and phenotypic data.

Full thickness uterine wall samples were obtained from deceased transplant organ donors after ethical approval (REC: 15/EE/0152, East of England–Cambridge South Research Ethics Committee) and informed consent from the donor families. The uterus was removed within 1 h of circulatory arrest.

### Donor inclusion criteria and endometriosis evaluation

Only individuals during their reproductive years were recruited and were considered as having ‘natural cycles’ only if they had not taken any hormonal treatment at least 3 months before sample collection. Donors with endometrial cancer were excluded. In addition, we aimed to exclude patients with other benign uterine/endometrial pathologies (that is, fibroids, polyps, adenomyosis, hyperplasia). However, in some cases (*n* = 15), later histological evaluations revealed the presence of these pathologies (Supplementary Table 1). Patients taking part in the ENDOX and FENOX studies (*n* = 69) were undergoing laparoscopic surgery for suspected endometriosis or infertility reasons at the John Radcliffe Hospital, Oxford. At the beginning of surgery, a pipelle biopsy of the endometrium was taken and the presence/absence of endometriosis, including endometriosis stage as per the revised American Society for Reproductive Medicine (rASRM stages I–IV), was assigned upon surgical evaluation during the laparoscopy. Four additional control samples (that is, samples from donors without endometriosis) came from the Sanger Cell Atlasing Project study (*n* = 3) and the Immunology of Subfertility study (*n* = 1). Absence of endometriosis was determined based on the clinical and medical history of the patients. For the Sanger Cell Atlasing Project, patients attended a coil clinic for contraceptive reasons. During the coil insertion procedure, a biopsy of the endometrium was taken in an outpatient setting. For the Immunology and Subfertility study, patients were undergoing in vitro fertilization and an endometrial biopsy was taken in an outpatient setting one cycle before the patient became pregnant and had a live birth.

### Assignment of menstrual stage

Optimal cutting temperature (OCT) blocks were sectioned at 10 µm thickness and hematoxylin and eosin (H&E)-stained following standard protocols. Menstrual phase was assigned based on histological evaluation by two independent pathologists. Where this was not possible, the menstrual phase was assigned based on the transcriptomic data and cellular profiles of the samples (Supplementary Table 1).

### Tissue processing

Superficial biopsies of the endometrium were collected using the Pipelle sampling device and immediately transferred into ice-cold PBS solution (Gibco, cat. no. 10010023). The endometrial tissue was then cut into smaller pieces and either moved into a cryovial and snap-frozen on dry ice (for single-nuclei extraction and processing) or moved into ice-cold HypoThermosol FRS solution (Sigma Aldrich, cat. no. H4416) and stored at 4 °C until further processing (either to be digested fresh or cryopreserved and digested later for single-cell processing). Where possible and sample size allowed, a small piece of tissue was also embedded in OCT compound (ThermoFisher Scientific, cat. no. 23730571) inside a cryomold and rapidly frozen in dry ice/isopentane slurry for histological evaluation and analyses.

Whole-uterus samples used for scRNA-seq and imaging analyses were stored in HypoThermosol FRS at 4 °C until processing. For imaging analyses, the samples were further dissected, embedded in OCT media and rapidly frozen in dry ice/isopentane slurry. For scRNA-seq (donor A70), to enrich endometrial cells, the endometrium was excised from the myometrium using scalpels and digested as detailed below.

Further details on tissue cryopreservation and dissociation for single cells/nuclei are described in Supplementary Note 8.

### H&E staining and imaging

Fresh frozen sections were removed from −80 °C storage and air-dried before being fixed in 10% neutral buffered formalin for 5 min. After rinsing with deionized water, slides were dipped in Mayer’s hematoxylin solution for 90 s. Slides were completely rinsed in 4–5 washes of deionized water, which also served to blue the hematoxylin. Aqueous eosin (1%) was manually applied onto sections with a pipette and rinsed with deionized water after 1–3 s. Slides were dehydrated through an ethanol series (70%, 70%, 100%, 100%) and cleared twice in 100% xylene. Slides were coverslipped and allowed to air dry before being imaged on a Hamamatsu Nanozoomer 2.0HT digital slide scanner.

### Multiplexed smFISH and high-resolution imaging

Large tissue section staining and fluorescent imaging were conducted largely as described previously^89^. Sections were cut from fresh frozen samples embedded in OCT at a thickness of 10 μm using a cryostat, placed onto SuperFrost Plus slides (VWR) and stored at −80 °C until stained. Tissue sections were then processed using a Leica BOND RX to automate staining with the RNAscope Multiplex Fluorescent Reagent Kit v2 Assay (Advanced Cell Diagnostics, Bio-Techne), according to the manufacturers’ instructions. Probes used are found in Supplementary Table 9. Before staining, tissue sections were post-fixed in 4% paraformaldehyde in PBS for 15 min at 4 °C, then dehydrated through a series of 50%, 70%, 100% and 100% ethanol, for 5 min each. Following manual pre-treatment, automated processing included epitope retrieval by protease digestion with Protease IV for 30 min before probe hybridization. Tyramide signal amplification with Opal 520, Opal 570 and Opal 650 (Akoya Biosciences), TSA-biotin (TSA Plus Biotin Kit, Perkin Elmer) and streptavidin-conjugated Atto 425 (Sigma Aldrich) was used to develop RNAscope probe channels. Stained sections were imaged with a Perkin Elmer Opera Phenix High-Content Screening System, in confocal mode with 1 μm z-step size, using a ×20 (numerical aperture (NA) 0.16, 0.299 μm per pixel) or ×40 (NA 1.1, 0.149 μm per pixel) water-immersion objective. Channels: DAPI (excitation 375 nm, emission 435–480 nm), Atto 425 (excitation 425 nm, emission 463–501 nm), Opal 520 (excitation 488 nm, emission 500–550 nm), Opal 570 (excitation 561 nm, emission 570–630 nm), Opal 650 (excitation 640 nm, emission 650–760 nm). Image stitching: confocal image stacks were stitched as two-dimensional maximum intensity projections using proprietary Acapella scripts provided by Perkin Elmer.

### 10x Genomics Chromium GEX library preparation and sequencing

Both cells and nuclei undergoing scRNA-seq and snRNA-seq were loaded according to the manufacturer’s protocol for the Chromium Single Cell 3′ Kit v.3.0 and v.3.1 (10x Genomics) to attain between 2,000 and 10,000 cells/nuclei per reaction. Libraries were sequenced, aiming at a minimum coverage of 50,000 raw reads per cell, on the Illumina Novaseq 6000 system, using the sequencing format: read 1: 28 cycles; i7 index: 10 cycles; i5 index: 10 cycles; read 2: 90 cycles.

### 10x Genomics Visium library preparation and sequencing

We generated 10x Genomics Visium transcriptomic slides from two superficial biopsies. Briefly, 10 μm cryosections were cut and placed on Visium slides v1 3′. These were processed according to the manufacturer’s instructions. Briefly, sections were fixed with cold methanol, stained with H&E and imaged on a Hamamatsu NanoZoomer S60 before permeabilization (20 min and 28 min), reverse transcription and complementary DNA synthesis using a template-switching protocol. Second-strand cDNA was liberated from the slide and single-indexed libraries prepared using a 10x Genomics PCR-based protocol. Libraries were pooled and sequenced on a Novaseq 6000, with the following sequencing format: read 1: 28 cycles; i7 index: 10 cycles; i5 index: 10 cycles; and read 2: 90 cycles.

### External human endometrial scRNA-seq and Visium datasets

We collected raw sequencing data from previously published human endometrial scRNA-seq datasets. Specifically, we downloaded publicly available .fastq files either from the Gene Expression Omnibus (GEO) or ArrayExpress. These datasets included: (1) Wang et al. (GEO accession number GSE111976)^14^, (2) Garcia-Alonso et al. (ArrayExpress accession numbers E-MTAB-10287 and E-MTAB-9260)^15^, (3) Tan et al. (GEO accession number GSE179640)^16^, (4) Lai et al. (GEO accession number GSE183837)^19^, (5) Fonseca et al. (GEO accession number GSE213216)^17^ and (6) Huang et al. (GEO accession number GSE214411)^18^. Only samples profiling eutopic endometrium from women during their reproductive years were included. Samples from endometriosis lesions or from menopausal women were excluded. We also collected scRNA-seq data from human cervical samples from the Genome Sequence Archive of the National Genomics Data Center (accession number PRJCA008573)^30^.

For spatial transcriptomics analysis, we used the 10x Genomics Visium from two full thickness uterus samples previously generated by us, available at ArrayExpress (accession number E-MTAB-9260).

### Alignment and quantification of scRNA-seq/snRNA-seq data

Reads from both the newly generated scRNA-seq/snRNA-seq libraries and external datasets were alignment to the 10x Genomics’ human reference genome GRCh38-2020-A, followed by cell calling, transcript quantification and QC using the Cell Ranger Software (v.6.0.2; 10x Genomics) with default parameters. Cell Ranger filtered count matrices were used for downstream analysis.

### Downstream scRNA-seq and snRNA-seq analysis

#### Donor demultiplexing and doublet identification

For 84 of the newly generated libraries (26 in the scRNA-seq and 58 in the snRNA-seq datasets) we multiplexed cell suspensions from two different donors. To ensure that we could confidently assign cells back to their donor, we genotyped some donors as described in Supplementary Note 9, and then pooled sample combinations in a way that each scRNA-seq and snRNA-seq library contained at least one genotyped donor.

To assign each cell/nucleus in the scRNA-seq and snRNA-seq libraries back to its donor-of-origin, we genotyped each barcode. Specifically, we called the single nucleotide polymorphisms (SNPs) in the reads from each barcode and piled them up using the cellSNP tool v.1.2.2 (ref. ^90^). Here, reads were genotyped from the Cell Ranger BAM files using a reference list of human common variants from the 1000 Genome Project (hg38 version with minor allele frequency > 0.0005) which we downloaded from https://sourceforge.net/projects/cellsnp/files/SNPlist. Once the cells in scRNA-seq and snRNA-seq libraries were genotyped, we linked them back to their donor-of-origin genotype (obtained using Illumina Global Array) using vireoSNP v.0.5.8 (ref. ^91^) with default parameters (n_donor = 2). Barcodes classified as either ‘doublet’ (that is, containing the two genotypes) or ‘unassigned’ were discarded in downstream analysis.

#### Doublet detection based on transcriptional mixtures

We quantified cell-doublet likelihood for each barcode with Scrublet software v.0.2.1 (ref. ^92^) on a per-library basis. We used a two-step diffusion doublet identification followed by Bonferroni false discovery rate correction and a significance threshold of 0.01, as described in ref. ^93^. Barcodes estimated as doublets were not excluded from the initial analysis, and instead these were kept in the downstream analysis and used to identify doublet-enriched clusters.

#### Quality filters, batch correction and clustering

For both scRNA-seq and snRNA-seq libraries, we used the filtered count matrices from Cell Ranger 6.0.2 for downstream analysis and analyzed them with Scanpy v.1.7.0 (ref. ^94^), with the pipeline following their recommended standard practices. We applied stringent QC to further filter the cells called by Cell Ranger to retain only high-quality cells. Specifically, we excluded cells either (1) expressing fewer than 1,000 genes or (2) with a mitochondrial content higher than 20%. For some datasets, these filters discarded more than 50% of the initial called cells.

Next, we flagged cell cycle genes using a data-driven approach as described in refs. ^93,95^. To do so, after converting the expression space to log(CPM/100 + 1), where CPM is counts per million, we transpose the object to gene space, performing principal component analysis (PCA), neighbor identification and Leiden clustering. The gene members of the gene cluster encompassing well-known cycling genes (*CDK1*, *MKI67*, *CCNB2* and *PCNA*) were all flagged as cell cycling genes, and discarded in each downstream analysis. In parallel, we also used the scanpy function ‘score_genes_cell_cycle’ to infer the cell cycle stage of each cell (that is, G1, G2/M or S) that was later used to interpret the clusters.

Next, we generated an integrated manifold for scRNA-seq and snRNA-seq datasets separately. The scRNA-seq manifold included data from six previously published studies as well as the scRNA-seq data newly generated by us. The snRNA-seq exclusively contains newly generated data for this study. To minimize cell cycle bias, the previously flagged cell cycle genes were excluded. The integrated manifolds were generated using single-cell Variational Inference (scVI) v.0.6.8 (ref. ^96^), with both the donor and study ID (that is, the dataset—for scRNA-seq only) as batches. All the remaining parameters were kept as default, with n_latent = 32, n_layers = 2. The scVI low dimensional space was estimated on the top 2,000 most highly variable genes in each dataset, which were defined using Seurat v3 flavor on the raw counts. With the resulting scVI-corrected latent representation of each cell, we estimated the neighbor graph, generated a uniform manifold approximation and projection (UMAP) visualization and performed Leiden clustering. The resolution of the clustering was adjusted manually so that all the previously described endometrial cell states^15^ were resolved.

The same integration strategy described in the paragraph above was used to reanalyse each of the four main cell lineages (that is, epithelial, mesenchymal, immune and endothelial) to further resolve the cellular heterogeneity in those compartments. Here, we subset the cells to those in the lineage and repeated scVI integration using the top 2,000 most highly variable genes within each lineage. The donor and the study ID were kept as batches, with default parameters, n_latent = 64 and n_layers = 2. The resulting scVI-corrected latent representations were used to derive per-lineage UMAPs and perform Leiden clustering. For the reanalysis of the immune compartment, donors taking exogenous hormones (Tan et al. dataset^16^) were excluded due to integration challenges.

### Annotation of cell types

We performed a full re-annotation of the cell clusters in the integrated scRNA-seq manifold. First, we carried out a new QC round to exclude clusters that were likely driven by technical artefacts (that is, low QC cells or doublets). Briefly, we flagged as low QC those clusters that (1) express an overall lower number of genes, (2) express an overall lower number of counts, (3) display a higher than average mitochondrial or nuclear RNA content and, importantly, (4) do not express any distinctive gene (and thus are not representing any independent biological entity). Next, we flagged as doublets those clusters that met the following criteria: (1) exhibit higher scrublet doublet score; (2) express marker genes from multiple lineages (for example, display both epithelial and immune markers) and (3) do not express any distinctive gene. Distinctive marker genes were identified using the Term Frequency–Inverse Document Frequency approach (TF-IDF), as implemented in the SoupX package v.1.5.0 (ref. ^97^).

Next, we assigned cell type labels to remaining high-quality clusters. General lineage annotation (that is, epithelial, mesenchymal, endothelial and immune) was done on the main manifold. Cell state annotation was inferred from the per-lineage manifold (that is, from reanalyzing the cells in each lineage, as described in the previous section), taking into account the following variables: (1) the menstrual cycle phase bias (or any other clinical variable such as exogenous hormones, endometriosis and so on), (2) the expression of previously described markers, (3) the differentially expressed genes and (4) the spatial location, either by performing smFISH or by deconvoluting the cellular composition of Visium spots. Cell type labels defined in the per-lineage manifold were then visualized on the general manifold.

Because of the higher gene coverage of the scRNA-seq data, cell type identification and annotation were done primarily on the integrated scRNA-seq dataset. To annotate the snRNA-seq clusters, we trained a Support Vector Machine (SVM) classifier (sklearn.svm.SVC) on the scRNA-seq dataset and transferred labels onto the denoized (that is, decontaminated of ambient RNA) snRNA-seq dataset. Denoising of snRNA-seq was done with DecontX from the R celda package v.1.6.1. Predicted cell type annotations on snRNA-seq were validated or disproved by looking at the expression of marker genes. Five samples underwent both single-cell and single-nuclei profiling and were further used as technical replicates to evaluate the agreement between nuclei–cell annotations.

To annotate immune cells in our datasets we first used celltypist v.0.1.9, which is a logistic regression classifier optimized by the stochastic gradient descent algorithm^98^. We trained the model by: (1) using both the ‘immune high’ and ‘immune low’ models built in to cell types and (2) next training our own model on the immune cells from the endometrial cell atlas^15^. After projecting labels from all three datasets, we refined the annotations using expression of bona fide markers on each cluster.

### Query-to-HECA mapping

We used the scArches model surgery framework^99^ to project new samples profiling human cervix from control donors onto the same latent space as single-cell HECA. See Supplementary Note 2.3 for further details.

### Alignment and quantification of Visium data

The newly generated 10x Visium spatial sequencing data were processed using Space Ranger Software (v.2.0.1) to identify the spots under tissue, align reads to the 10x Genomics human reference genome GRCh38-2020-A and quantify gene counts. Spots were automatically aligned to the paired H&E images by Space Ranger software. All spots under tissue detected by Space Ranger were included in downstream analysis.

### Downstream analysis of Visium data

#### Location of cell types in Visium data

We spatially mapped the cell types from the scRNA-seq dataset on the Visium slides with cell2location tool v.0.06-alpha (ref. ^100^). We deconvoluted both the Visium slides newly generated in this study from superficial biopsies and the ones downloaded from E-MTAB-9260 covering full thickness uterus. As reference, we used the cell type signatures from the scRNA-seq dataset, subsetting the cells to those expressing more than 2,000 genes. Cell2location was run with default parameters, with the exception of cells_per_spot which was set to 20. Each Visium section was analyzed separately. The estimated abundance for each cell type was visualized following the cell2location tutorial.

### Cell–cell communication analysis with CellPhoneDB

Because two cell types can only interact paracrinally or juxtacrinally if they co-localize in space and time, we first manually classified the cell types into the spatiotemporal microenvironments where they coexist (for example, endothelial and PV cells coexist in the vessels, while preGlandular cells coexist with dStromal early cells in the functionalis layer of the early secretory endometrium). Spatial location was derived from previous knowledge, smFISH experiments or cell type deconvolution of Visium spots with cell2location. The temporal location was directly derived from the menstrual phase where the cell types are detected.

To identify paracrine or juxtacrine interactions between the cells co-localizing in an endometrial microenvironment, we used the differentially expressed genes (DEGs)-based method of CellphoneDB v.4.0.0 (ref. ^101^). Using this method, we retrieved interacting pairs of ligands and receptors meeting the following requirements: (1) all the interacting partners were expressed by at least 10% of the cell type under consideration; (2) the interacting cell type pairs share an endometrial microenvironment and (3) at least one of the interacting partners (for example, either the ligand or the receptor) was significantly upregulated in the corresponding cell state of a lineage (Wilcoxon tests; adjusted *P* < 0.01 and a log_2_ fold change > 0.75). Differential expression analysis was performed on a per-lineage basis to identify the genes specifically upregulated in a cell state compared with the other cell states in the same lineage. Donors under exogenous hormonal therapy were excluded from the analysis. These interactions between HECA cell types can be iteratively queried via the CellPhoneDBViz browser at https://www.reproductivecellatlas.org.

The interactions identified were further tested by the LIANA+ (ref. ^102^) tool. LIANA+ uses an integrative database of ligand–receptor interactions (including the CellphoneDB database) and computes a combined score by ranking and aggregating the ligand–receptor interaction prediction from multiple statistical frameworks (including the generic CellPhoneDB statistical analysis). We ran LIANA+ on each endometrial microenvironment, set specificity_rank ≤ 0.2 as our significance threshold and report the validated cellular interactions in Supplementary Table 4. The full table of interactions retrieved by LIANA+ can be found at https://github.com/ventolab/HECA-Human-Endometrial-Cell-Atlas/tree/main/cellphoneDB.

### Reporting summary

Further information on research design is available in the Nature Portfolio Reporting Summary linked to this article.

## Online content

Any methods, additional references, Nature Portfolio reporting summaries, source data, extended data, supplementary information, acknowledgements, peer review information; details of author contributions and competing interests; and statements of data and code availability are available at 10.1038/s41588-024-01873-w.

## Supplementary information

Supplementary Information Supplementary Notes 1–9. 1. Datasets integration and comparison. 2. Computational resources from the HECA. 3. Annotation of the epithelial cells. 4. Annotation of the stromal cells. 5. Differential cell abundance (DCA). 6. Cell type enrichment analysis for endometriosis GWAS genes. 7. Differential gene expression endometriosis versus controls. 8. Tissue processing and cryopreservation. 9. Donor genotyping.

Reporting Summary

Supplementary Tables 1–9 Supplementary Table 1. Harmonized metadata of samples analyzed. Supplementary Table 2. Cell Ranger QC outputs for all newly generated data. Supplementary Table 3. Absolute cell counts per donor and dataset in the HECA. Supplementary Table 4. Cell–cell interactions identified using LIANA. Supplementary Table 5. Differentially expressed genes reported for stromal cells between controls and endometriosis cases. Differential expression analysis was performed using limma v.3.54.2. The table reports the magnitude of differential expression (log_2_ fold change) and false discovery rate-adjusted *P* values using the Benjamini–Hochberg method. Supplementary Table 6. Differentially expressed genes reported for macrophages between controls and endometriosis cases. Differential expression analysis was performed using limma v.3.54.2. The table reports the magnitude of differential expression (log_2_ fold change) and false discovery rate-adjusted *P* values using the Benjamini–Hochberg method. Supplementary Table 7. Reagents used for the snRNA-seq homogenization buffer. Supplementary Table 8. Reagents used for the snRNA-seq wash buffer. Supplementary Table 9. List of smFISH probes used for smFISH imagining.

## Data Availability

Datasets are available from ArrayExpress (www.ebi.ac.uk/arrayexpress), with accession numbers E-MTAB-14039 (scRNA-seq and snRNA-seq) and E-MTAB-14058 (Visium spatial transcriptomics). Multiplexed smFISH images are available from BioStudies (www.ebi.ac.uk/biostudies), with accession number S-BIAD1182. All data are available for public access. scRNA-seq and snRNA-seq datasets to reproduce UMAPs and dot plots can be accessed and downloaded through the web portal: https://www.reproductivecellatlas.org/endometrium_reference.html.

## Code availability

All the code used for data analysis is available at: https://github.com/ventolab/HECA-Human-Endometrial-Cell-Atlas.
