# Supplementary material for: An integrated single-cell reference atlas of the human endometrium
Source: Nat Genet. 2024 Aug 28;56(9):1925–37. doi: 10.1038/s41588-024-01873-w (PMC11387200; doi:10.1038/s41588-024-01873-w)
Supplement: Supplementary file 1 — Supplementary Notes 1–9. 1. Datasets integration and comparison. 2. Computational resources from the HECA. 3. Annotation of the epithelial cells. 4. Annotation of the stromal cells. 5. Differential cell abundance (DCA). 6. Cell type enrichment analysis for endometriosis GWAS genes. 7. Differential gene expression endometriosis versus controls. 8. Tissue processing and cryopreservation. 9. Donor genotyping. [file 41588_2024_1873_MOESM1_ESM.pdf]

nature genetics

Supplementary information https://doi.org/10.1038/s41588-024-01873-w

An integrated single-cell reference atlas of the human endometrium

In the format provided by the authors and unedited

Table of contents

Supplementary Notes **2**
Supplementary Note 1. Datasets integration and comparison 2
1.1 Integration and identification of cell states 2
1.2 Datasets 5
1.2.1 Single-cell RNA-seq datasets in HECA 5
1.2.2 Single-nuclei RNA-seq validation dataset 5
1.3 Datasets comparison 6
1.3.1 Across scRNA-seq datasets 6
1.3.2 scRNA-seq versus snRNA-seq 7
Supplementary Note 2. Computational resources from the HECA. 9
2.1 Atlas visualisation and query with CellxGene 9
2.2 Cell-cell communication visualisation and query with CellPhoneDBViz 9
2.3 Aligning new datasets onto HECA 9
2.3.1 scANVI model weights 9
2.3.2 Query-to-HECA mapping: scArches tutorial 10
Supplementary Note 3. Annotation of the epithelial cells. 11
Supplementary Note 4. Annotation of the stromal cells. 16
Supplementary Note 5. Differential cell abundance (DCA). 18
Supplementary Note 6. Cell type enrichment analysis for Endometriosis-GWAS genes. 19
Supplementary Note 7. Differential gene expression endometriosis vs controls. 19
Supplementary Note 8. Tissue processing and cryopreservation. 20
8.1. Tissue cryopreservation 20
8.2. Tissue dissociation for single-cell RNA-sequencing 20
8.3. Tissue dissociation for single-nucleus RNA-sequencing 20
Supplementary Note 9. Donor genotyping. 21
Supplementary References **22**

Supplementary Notes

Supplementary Note 1. Datasets integration and comparison

Here we have assembled the first consensus cell atlas of the endometrium, the Human Endometrial Cell Atlas (HECA). The HECA maps the distinct stages of the menstrual cycle in women of reproductive age in both controls (i.e. donors without reported endometrial/uterine pathologies) and donors with endometriosis (**Supplementary Table 1**). The analysed samples were from either donors having natural menstrual cycles or from donors taking exogenous hormones for either contraceptive reasons or as a treatment for endometriosis. By adding donors with and without endometriosis as well as donors taking exogenous hormonal regimes, the HECA can be used as a “reference” map covering the most common clinical characteristics observed in the real population world-wide.

1.1 Integration and identification of cell states

To assemble the HECA, we integrated single-cell RNA sequencing (scRNA-seq) data generated and published by multiple teams, including ours (see **Supplementary Note 1.2** below and **Supplementary Tables 1-2**). The previously published smaller-scale studies have so far lacked consensus cell state annotation and reproducible marker gene signatures across atlases. Generally, these studies cover individuals with distinct - often non-overlapping - clinical characteristics: while certain studies focus exclusively on profiling cells from either control individuals or endometriosis patients, others consider donors taking exogenous hormonal therapy. Also, the datasets looking at endometriosis cases do not often include controls covering the same menstrual stages as the endometriosis patients, especially along the highly dynamic secretory phase. This lack of harmonisation between datasets challenges the comparison across studies, and thus, the relationship of the cell populations in the different atlasses is unknown. Additionally, the different studies have used distinct strategies to dissociate the tissue into single cells (see **Supplementary Table 1** and Table **1.2.1** below), and the potential impact this may have on the transcriptomic profiles and cell type recovery remains unexplored.

To evaluate the impact of both biological and technical variables, we first generated a cell-level atlas integrating the transcriptomic profiles of 6 previously published scRNA-seq datasets (i.e. the Wang et al.[1], Garcia-Alonso et al.[2], Tan et al.[3], Lai et al.[4], Fonseca et al. [5], and Huang et al. [6] datasets). We also generated a new single-cell dataset (i.e. the “Mareckova cells” dataset; **Supplementary Table 2**) for a set of donors that share similar clinical characteristics with the six previously published studies, overcoming the limited overlap of clinical variables across the existing datasets. We used the “Mareckova cells” dataset as an integration ‘anchor’ that allowed us to assess the impact of both biological and technical variables on the integration process. In addition, the Mareckova cells dataset contains biological replicates, allowing us to further evaluate our batch correction strategy - i.e. select the best method that preserves biological signatures while correcting for dataset and/or donor-specific signatures.

Altogether, the integrated single-cell level atlas consists of ~314,000 cells from 63 individuals during natural menstrual cycles (n=49), taking exogenous hormones at sample collection

(n=14) and with/without endometriosis (**Fig. 1b-c, Supplementary Table 1**). The majority of the samples profiled are from superficial biopsies of the endometrium, predominantly sampling the functionalis layer from living donors (n=60). It also includes 3 samples/biopsies of full-thickness endometrium (i.e. samples containing both the functionalis and basalis layers) with attached subjacent myometrium from the uteri of donors who died of non gynaecological causes. Including these full-thickness samples allowed us to explore both the functionalis and basalis layers of the endometrium.

To validate the findings of the single-cell level atlas, we generated an independent single-nuclei RNA sequencing (snRNA-seq) dataset (i.e the Mareckova nuclei dataset; **Supplementary Table 2**) from a collection of snap-frozen samples from individuals presenting with the same spectrum of clinical characteristics (e.g. controls, endometriosis cases, individuals taking exogenous hormones at sample collection, etc.). In comparison to scRNA-seq, snRNA-seq data captures nuclear RNA only and contains higher levels of ambient RNA (i.e. 'soup') resulting from the extraction protocol that bursts cells to release the nuclei. The higher presence of ambient/soup RNA can potentially interfere with the analysis, as it might lead to the detection of gene expression that are actually released from the cytoplasm of a different cell type. However, the advantage of snRNA-seq is in its use in profiling archival frozen samples and that, in this study, the snRNA-seq dataset did not contain batch effects occurring as a result of various digestion protocols used for scRNA-seq. As such, it is a unique dataset to validate our findings of the scRNA-seq data analyses. Importantly, five samples were in parallel processed for both single cell (cryopreserved) and single nuclei (snap-frozen) profiling and were used as technical replicates to evaluate the alignment between the single-cell and single-nuclei datasets. This single-nuclei atlas consists of ~312,000 nuclei from 63 individuals during natural menstrual cycles (n=47), taking exogenous hormones at sample collection (n=16) and with/without endometriosis, all from superficial endometrial biopsies (**Fig. 1b and d, Extended Data Fig. 4** and Table **1.2.2** below).

Technical details on the integration strategy are described in the **Methods** section at "Downstream sc/snRNA-seq analysis" > "Quality filters, batch correction and clustering". Briefly, integration was performed on the scRNA-seq and snRNA-seq atlases separately with single-cell Variational Inference (scVI)[7] v0.6.8. To generate the cell-level HECA, we considered the "dataset id" as the batch. The inclusion of the Marekova-cell dataset ensures that the relevant clinical variables are represented by at least two datasets so that scVI can account for and remove dataset-specific signatures but preserve the biological signatures related to clinical variables. scVI generates a dataset-corrected latent space from the uncorrected raw gene counts, which is used for Uniform Manifold Approximation and Projection (UMAP). Thus, corrections are not applied at the gene-level meaning that downstream tests done at the gene-level remain unaffected by batch removal, and the gene expression illustrated in the dotplots along this manuscript is not corrected for batch effects. Integration and clustering resolution were considered satisfactory when well-known cell types from different datasets were aligned on the UMAP space. Definition and annotation of cell types relied on the expression of bona fide markers (for previously described populations) or newly identified markers (for the previously unreported populations), with the markers remaining consistent across the datasets and donors to ensure that these are not dataset-specific (see "Annotation of cell types" in **Methods**).

The reported cell types were validated at the transcriptomic level through:

I. Individual and integrated analysis of each scRNA-seq dataset. Detecting the same cell subset in the individual (i.e. per donor) and integrated manifold confirmed the robustness of the identified cell states across datasets.
II. Analysis of the independent Mareckova snRNA-seq dataset. The snRNA-seq does not contain batch effects introduced when data is generated by different laboratories and/or cell-dissociation techniques. The majority of cell states identified by the single-cell HECA were identified in the scRNA-seq datasets. See **Supplementary Note 1.3.2** below for a further discussion on "scRNA-seq versus snRNA-seq comparison".
III. Integration of spatial transcriptomics (Visium) with scRNA-seq data. This approach allowed us to determine the in situ spatial locations of the identified cell states. This strategy's strength lies in using whole transcriptomics information rather than specific markers to map cells in space. This is especially relevant for transitioning cell states that are identified based on marker gene regulation dynamics rather than exclusive marker gene expression. In addition, the findings were further supported by smFISH using distinctive markers.

1.2 Datasets

1.2.1 Single-cell RNA-seq datasets in HECA

Dataset	Menstrual stages	Donors taking exogenous hormones	Disease*	Digestion protocol
Mareckova (cells)	Proliferative, Secretory & Menstrual	- & +	Endometriosis & controls	Collagenase V & Trypsin
Wang	Proliferative & Secretory	-	Controls	Collagenase A1 & TrypLE
Garcia-Alonso	Proliferative & Secretory	-	Controls	(i) Collagenase V & Trypsin *or* (ii) Collagenase V only
Lai	Secretory (WOI)^	-	Controls (donors with recurrent implantation failure are not included in the HECA)	Collagenase IV
Tan	Proliferative & Menstrual	- & +	Endometriosis & controls	Cold active caspase & Dispase
Fonseca[+]	Proliferative & Secretory	- & +	Endometriosis	Collagenase/Hyaluronidase
Huang	Proliferative & Secretory	-	Endometriosis & controls	Collagenase IV

^ WOI, window of implantation
** Controls refers to donors without endometriosis*
+ Menopause donors were excluded

1.2.2 Single-nuclei RNA-seq validation dataset

Dataset	Menstrual stages	Donors taking exogenous hormones	Disease*	Digestion protocol
Mareckova (nuclei)	Proliferative, Secretory & Menstrual	- & +	Endometriosis & controls	Mechanical homogenisation

** Controls refers to donors without endometriosis*

1.3 Datasets comparison

1.3.1 Across scRNA-seq datasets

We observed striking differences in cell type composition of the different datasets with respect to the 4 main cell lineages: epithelial, stromal, mesenchymal and immune **(Fig. 1e, Supplementary Table 3)**. For example, the Fonseca et al. and Lai et al. datasets consisted of ~5% and <1% epithelial cells, respectively. The Wang et al. dataset consisted of the highest proportion of epithelial cells (~60%), followed by the Mareckova et al. nuclei dataset (~48%). The datasets capturing mostly mesenchymal cells were the Lai et al. and Fonseca et al. with ~90% and 85%, respectively, followed by Garcia-Alonso et al. consisting of ~73% mesenchymal cells. The lowest proportion of endothelial cell recovery was noted for the Lai et al. dataset (<1%) and the Mareckova et al. nuclei dataset (~1%). With regards to the immune cell lineage, the Fonseca et al. dataset had the lowest number of immune cells detected (~1%) while the Tan et al. dataset consisted of ~28% of immune cells, which was the highest proportion of immune cells observed per dataset. These observed differences in the recovery of the different cell populations among the datasets may have been caused by the choice of tissue digestion protocol, sampling bias of different endometrial regions, stage of menstrual cycle and use of exogenous hormones **(see Methods, Extended Data Fig. 1, Supplementary Table 1)**. For example, samples generated using a protocol including a trypsin digestion step display the highest proportion of epithelial cells (i.e. Wang and Mareckova datasets; **Fig. 1e** and **Supplementary Note 1.2**), although additional factors exist as Huang et al. protocol lacking the trypsin step recovers ~20% epithelial cells in their study.

With regards to the effect of exogenous hormones on the transcriptomic profile of cells analysed, we noted large variation between cell clusters identified and the hormones taken at sample collection **(Extended Data Fig. 5)**. Exogenous hormones are routinely prescribed as a contraceptive means as well as treatment for endometrial/uterine pathologies and include commonly used contraceptives. A plethora of contraceptive options exists (e.g. the pill, intrauterine devices, etc.), with different hormonal formulations and modes of administration having different effects on the endometrium and its morphology[8,9]. In line with that, we noted that, especially, the epithelial cells from donors on hormones were considerably different to epithelial cells obtained from donors during natural menstrual cycles **(Fig. 1c and d)**, forming their own cell clusters specific to certain drug formulations **(Extended Data Fig. 5)**. Due to the low number of samples per each hormonal treatment and a lack of a thorough clinical metadata collection, these clusters were grouped under one ‘Hormones’ cluster. As individuals with endometriosis are often prescribed and take exogenous hormones, future studies should focus on obtaining further clinical information, such as the patient's ovulation status, length and formulation of treatment taken and detailed histological description if patients on hormones are to be included and compared to controls. The treatment must be matched and in a large enough sample size in order to draw any conclusions from the comparisons made.

Due to the above described differences observed, samples from donors on hormones are not included in any downstream analyses - they are only used to assemble the HECA. As such, no conclusions drawn in our manuscript are undermined nor influenced by samples from donors on hormones. The inclusion of these samples to assemble the HECA was a

deliberate choice, allowing us to highlight and convey the following important messages:

1. Endometrial cells from donors taking exogenous hormones have a different transcriptomics profile, a finding previous studies (e.g. Fonseca et al., Tan et al.) failed to describe and address, leading to cells being mislabelled. By assembling the HECA using cells from donors with and without hormones, we were able to (a) define what the differences and commonalities are between the two cell sources, (b) harmonise cell labelling across datasets and (c) make a case for more careful annotation of endometrial cells from donors taking exogenous hormonal therapy.

2. The effect of exogenous hormones is more profound in the epithelial lineage, while stromal cells from donors on hormones (with some exceptions) appear similar to stromal cells from donors not taking exogenous hormonal therapy (except donor specific clusters). Finding confirmed also by the single-nuclei data.

3. The use of different hormonal regimens has a profound effect on cells' transcriptomic profiles, requiring additional metadata collection and profiling of a larger sample size by future studies before any conclusions can be drawn about the effects of each treatment on cells' transcriptome (see above). In addition, the data shown in **Extended Data Fig. 5** showcase the transcriptomic heterogeneity of various hormonal regimens very well, prompting future researchers to carefully consider their study designs and data integration approaches. Undoubtedly, the HECA assembled here will provide a key framework to resolve the transcriptomics heterogeneity and further our understanding of the effects of exogenous hormones on cellular transcriptomes.

Altogether, we hope that by adding donors with and without endometriosis as well as donors taking exogenous hormonal regimes, the HECA can be used as a "reference" map covering the most common clinical characteristics observed in the real population that includes millions of women world-wide taking exogenous hormones either for contraceptive reasons or as treatment options for a multitude of reproductive conditions.

1.3.2 scRNA-seq versus snRNA-seq

While most cell states identified in the scRNA-seq dataset were successfully validated and detected in the snRNA-seq dataset, a few exceptions were observed: (i) Glandular secretory (FGF7+) epithelial cell population: this cell subset appears in late secretory stage samples, but was not detected by snRNA-seq. As the endometrium is highly dynamic and undergoes dramatic changes across the cycle, it is likely that samples spanning the same stage/timepoint of the late secretory stage were not included in the nuclei dataset. (ii) KRT5 epithelial population: this population is present in the cervix and was not present in all scRNA-seq samples and not detected by snRNA-seq at all. It is a contaminant present due to the nature of how superficial endometrial biopsies are collected - by first passing the Pipelle sampler through the cervix. It is likely such cervical cell contaminants were not present in the biopsies taken for snRNA-seq analyses. (iii) Hormone clusters: in the HECA we resolved 3 main clusters - cluster 16 for epithelial cells and clusters 30 and 31 for stromal cells. As donors were taking various types of exogenous hormones at sample collection, we did not resolve further heterogeneity within these three main clusters, but provide details on

cellular composition for each hormonal treatment in **Extended Data Fig. 5**. With regards to cluster 16 (i.e. Hormones cluster for epithelial cells), we detect nuclei with similar signatures also in the snRNA-seq dataset. However, for clusters 30 and 31 (i.e. Hormones clusters for stromal cells), we do not detect nuclei with such transcriptomic signatures in the snRNA-seq dataset. Cluster 31 is a donor-specific cluster in the scRNA-seq dataset/HECA, which can explain its absence in the snRNA-seq dataset. Cluster 30 contains cells from multiple donors in the scRNA-seq dataset/HECA, predominantly 3 donors from the Tan et al. dataset taking the same hormonal treatment (i.e. norethindrone plus oestrogen). In the snRNA-seq dataset, no donors were taking such treatment, which can explain the absence of such a cell cluster in the snRNA-seq dataset. (iv) mPV. A perivascular cell subset found in the myometrium, detected in full-thickness endometrial biopsies from the whole uterus. As the nuclei data is derived only from superficial endometrial biopsies, this population is absent in the nuclei dataset.

In addition, we observed a lower recovery rate for perivascular (PV) and endothelial cells in the single-nuclei dataset when compared to the single-cell datasets **(Fig. 1e)**. Multiple factors might be responsible for this observation, including the way in which the endometrial samples are dissociated to obtain single cells and single nuclei. In most cases, a two-step enzymatic digestion protocol is used to dissociate the tissue for scRNA-seq, suggesting that in the initial step the collagenase enzyme used readily releases the stromal, PV and endothelial cells from the extracellular matrix while the epithelial glands remain to some extent undigested and require further treatment with trypsin. Due to this, the proportion of stromal, PV and endothelial cells may be higher in scRNA-seq data. It may also be that the nuclei extraction protocol is not fully optimised for capturing PV and endothelial nuclei or that snap-freezing of the samples bursts and selectively damages the nuclei of endothelial and perivascular cells.

Supplementary Note 2. Computational resources from the HECA.

In order to facilitate the use of the HECA by the research/scientific community, we provide the full annotated dataset, interactive visualisation tools and pretrained model weights for integration with new/other external datasets. These can be accessed at https://www.reproductivecellatlas.org/endometrium_reference.html.

2.1 Atlas visualisation and query with CellxGene

Chan Zuckerberg CELL by GENE (CZ CELLxGENE) is an online platform which supports interactive visualisation and exploration of single cell datasets. We provide interactive objects for: (i) the full HECA (cells), (ii) the full single-nuclei dataset, (iii) immune cells only and (iv) immune nuclei only, where users can interactively explore the distribution of different covariates and/or the expression of any gene of interest. Detailed tutorials of how to query these atlases can be found at https://cellxgene.cziscience.com/docs/04__Analyze%20Public%20Data/4_1__Hosted%20Tutorials

2.2 Cell-cell communication visualisation and query with CellPhoneDBViz

CellphoneDBViz is a software for visualising the results of either differential or statistical analyses for cell-cell communication by the CellphoneDB package (https://github.com/ventolab/CellphoneDB), version >= 4.0.0 only. The software accepts input to and output files from a CellphoneDB analysis as well as a simple configuration file in YAML format. From the files included in the configuration file, CellphoneDB Viz first works out automatically which version of the software was used and which CellphoneDB analysis was run, and then includes the relevant plots, filters and legends accordingly.

The software consists of a web server, an API that serves the data to the front end, and the html including JavaScript code that fetches the data from the API and visualises it in various plots. The plots have been implemented mostly in D3 (https://d3js.org/). Examples of visualisations produced via CellphoneDB Viz can be found in https://www.cellphonedb.org/viz/index.html and the full package documentation is included in https://github.com/datasome/cellphonedbviz/blob/main/README.md.

2.3 Aligning new datasets onto HECA

2.3.1 scANVI model weights

In single-cell genomics, mapping new datasets to large, high quality atlases helps leverage the information in these reference atlases to contextualise new observations. The computational framework scArches[10] uses transfer learning to efficiently update an existing model (trained on a reference dataset) and integrate new datasets.

To enable mapping of new data to the HECA, we trained a scANVI model directly on the final version of the HECA. scANVI is a probabilistic model which builds on the scVI framework. Like scVI, it uses a variational autoencoder to learn a lower dimension representation of each cell. scANVI also leverages information from cell type annotations to improve data

integration and model performance. In order to build a model compatible with the scArches framework, we trained a scANVI model with only sample ID as batch covariate (note that the scVI model used in main analysis was trained using sample ID and dataset as covariates). We trained the reference scANVI model for 20 epochs, based on an scVI model with n_layers=2. The surgery model was trained for 100 epochs with weight_decay=0.0 to ensure reference cell embeddings would remain identical. To obtain joint embeddings, we concatenated gene expression counts from HECA reference cells and query samples into a single object and used the surgery encoder to get latent representations. We then computed the kNN graph (default parameters) and UMAP (min_dist=0.4) on the joint embeddings. We evaluated the quality of the query to reference mapping by examining the alignment on the UMAP and the concordance of marker gene expression in HECA reference cells and query samples. The scANVI model trained on HECA is available on the Reproductive Cell Atlas portal at https://www.reproductivecellatlas.org/endometrium_reference.html.

2.3.2 Query-to-HECA mapping: scArches tutorial

We provide step-by-step scArches (v0.5.9) tutorials at https://github.com/ventolab/HECA-Human-Endometrial-Cell-Atlas/blob/main/tutorials/query_to_ref_mapping.ipynb to support mapping any new samples to the HECA reference cells based on any input gene expression count matrix.

Supplementary Note 3. Annotation of the epithelial cells.

To annotate the epithelial cell states present in the human endometrium we considered: (i) the distinctive expression of genes, including bona fide markers (**Fig. 2a & Fig. 3a**), (ii) the menstrual stage at which these cells appear (**Fig. 1f**), and (iii) their spatial coordinates, as inferred by integrating single-cell transcriptomics with Spatial Transcriptomics (Visium) (**Fig. 2b-f** and **Extended Data Fig. 7**). Altogether, we resolved the following cell states:

Cell type	Cell state	Markers and distinctive genes	Menstrual stages	Location
SOX9+	SOX9 basalis (CDH2+)	*SOX9, MMP7, FOXA2, CDH2hi, AXIN2, TRH, ALDH1A1, KLK11, IHH, EMID1*	Proliferative & Secretory*	Basalis glands
SOX9+	SOX9 functionalis I (CDH2+)	*SOX9, MMP7, FOXA2, CDH2, TRH, SLC7A11, DKK1, PHLDA1, KMO, IHH, EMID1*	Proliferative	Functionalis glands
SOX9+	SOX9 functionalis II	*SOX9, MMP7, FOXA2, CDH2low,TRH, KMOhi, IHHhi, EMID1hi*	Proliferative	Functionalis glands
SOX9+	SOX9 luminal (LGR5+)	*SOX9, MMP7, IL32, WNT7A, LGR5,TNF*	Proliferative	Functionalis lumen
Glandular	preGlandular	*FOXA2, ABCG1, HPRT1, SUFU, OPRK1, CBR3*	Secretory - Early	Functionalis glands
Glandular	Glandular	*FOXA2, ABCG1, S100P, SCGB2A2, PAEPlow*	Secretory - Mid	Functionalis glands
Glandular	Glandular secretory	*FOXA2, ABCG1, PAEPhi, DPP4, GPX3*	Secretory - Mid	Functionalis glands
Glandular	Glandular secretory (FGF7+)	*FOXA2low, ABCG1low, PAEPhi, DPP4, GPX3, PTPRR, FGF7, FXYD2*	Secretory - Late	Functionalis glands
Luminal	preLuminal	*LGR5, VTCN1, CLDN22, SULT1E1*	Secretory - Early	Functionalis lumen
Luminal	Luminal	*LGR5, VTCN1hi, CLDN22, SULT1E1low, LEFTY1hi, LGR5, PTGS1hi, IL6hi*	Secretory - Mid and late	Functionalis lumen
Ciliated	preCiliated	*CCNO, CDC20B, MUC12, PIFO, FOXJ1, TP73*	Proliferative	Functionalis glands & lumen
Ciliated	Ciliated	*PIFO, FOXJ1, TP73*	Secretory	Functionalis glands & lumen
Endocervix/Cervix /	MUC5B	*BPIFB1, TFF3, MUC5B, SAA1*	Proliferative & Secretory	Endocervix/Cervix & *MUC5B* gene expression also in endometrium by smFISH
Endocervix/Cervix	KRT5	*TP63, SNCG, KRT5*	Proliferative & Secretory	Endocervix/Cervix

Abbreviations: hi, high expression; low, low expression; smFISH, small-molecule fluorescent in situ hybridisation.

**Subset found in the secretory phase detected by spatial transcriptomics (Visium & smFISH) but absent in the secretory phase of scRNA-seq and snRNA-seq likely due to sampling bias.*

<u>*SOX9*+ cells:</u>

SOX9 belongs to a family of Sry-related HMG box (SOX)-containing transcription factors and is involved in many developmental processes including neural crest specification, chondrogenesis and gonadal sex differentiation[11]. In particular, *SOX9* is expressed in the stem/progenitor cell compartment in both the branching lung tips and intestinal crypts[12,13] and plays a crucial role in regulating progenitor proliferation in the lungs[14] or in the intestinal stem cell niche supporting the neighbouring stem cells[15,16]. Importantly, *SOX9* can be upregulated by β-Catenin-dependent canonical WNT signalling[13,14]. This is consistent with our observation that WNT components are highly involved in all the *SOX9*+ populations and suggests that cells with stem/progenitor activity may reside within these *SOX9*+ populations.

- We report a population of **SOX9 basalis (*CDH2+*)** epithelial cells expressing markers previously described for endometrial epithelial stem/progenitor cells such as *CDH2, AXIN2, ALDH1A1*[17–19] and also defined by the expression of *TRH, KLK11, IHH,* and *EMID1*. This cell population was mapped to the basalis layer of the endometrium using spatial transcriptomics and smFISH in both the proliferative and secretory samples. We also identified two *SOX9*+ populations mapping to the functionalis glands in proliferative phase endometrium, which we name SOX9 functionalis I (expressing *CDH2, SLC7A11, DKK1, PHLDA1, TRH, KMO, IHH, EMID1*) and SOX9 functionalis II (expressing *CDH2low,TRH, KMOhi, IHHhi, EMID1hi*).

  CDH2 (encoding N-cadherin) has many cellular context-dependent roles ranging from cell-cell adhesion, mediating EMT and linking the actin cytoskeleton via a cadherin/catenin complex[20,21]. The interaction of N-cadherin with β-Catenin effectively modulates the level of WNT/β-Catenin signalling by sequestration, however the presence of proteinases such as MMPs can cleave N-cadherin and release β-Catenin for nuclear localisation and signal transduction[22]. Furthermore, *AXIN2* is a direct target of β-Catenin-dependent canonical WNT signalling[23,24], suggesting active WNT/β-Catenin signalling to maintain the putative SOX9 basalis (*CDH2*+) epithelial stem/progenitor cells in the basalis layer of the endometrium. In line with this, high WNT signalling is crucial to maintain the stem/progenitor cell niche in the small intestinal crypts [25] and in the developing lung progenitors[26]. The SOX9 functionalis I and II population express lower levels of *CDH2* and *AXIN2* than the SOX9 basalis (*CDH2*+) population, which could be explained by the upregulation of the WNT pathway inhibitor *DKK1* in the SOX9 functionalis I cells. SOX9 functionalis I and II are likely differentiated from the SOX9 basalis (*CDH2*+) epithelial stem/progenitor cell population and give rise to the functionalis glandular cells.

- The **SOX9 functionalis I and II** populations express higher levels of *IHH* in comparison to the putative SOX9 basalis (*CDH2*+) epithelial stem/progenitor cells. *IHH* is part of Hedgehog signalling, which has been involved in regulating stem cell proliferation and/or differentiation in a cell type-specific context[27], and has further been shown to be required for endometrial remodelling in mice[28]. Hedgehog signalling also shares signalling components with the WNT pathway such as GSK3 and has been proposed to interact with the WNT signalling agonistically or antagonistically in the small intestine or colon respectively[27,29]. The SOX9 functionalis I population also expresses genes that are targets of oestrogen receptor signalling,

such as *PHLDA1*, indicating a response of these cells to the oestrogen present in the proliferative phase endometrium[30].

- In the lumen of the proliferative phase, we also define a **SOX9 luminal (*LGR5*+)** population in agreement with our previous reports[2]. It is characterised by the expression of members of the WNT signalling pathway, including *LGR5* and *WNT7A*. However, *AXIN2,* which is a direct target of the canonical WNT signalling pathway, is not upregulated, implying that the WNT signalling in this population may operate through the non-canonical WNT pathway. Consistent with this notion, WNT7A is known to activate the non-canonical pathway in other tissues[31,32]. Although *LGR5* expression is primarily involved in enhancing canonical WNT/β-Catenin signalling in the presence of R-spondins[33,34], its expression nonetheless marks long-lived adult stem cells in several tissues[35]. The SOX9 luminal (*LGR5*+) population also expresses inflammatory markers (*IL32*, *TNF*), in line with the upregulation of pro-inflammatory signals in response to oestrogens.

All four populations express *SOX9* with this marker gene most strongly upregulated in the SOX9 functionalis I population.

Glandular cells

Within the *functionalis* glands of the secretory phase we resolved the preGlandular (early secretory phase), Glandular, Glandular secretory (mid secretory phase) and Glandular secretory (*FGF7*+; late secretory phase).

- The **preGlandular** population is marked by the downregulation of Hedgehog signalling (e.g. *SUFU*[36,37] and the establishment of apico-basal polarity (*CRB3* reviewed in[38]).

- The **Glandular** population is characterised by the expression of genes involved in the formation of glandular structures such as *SCGB2A2* and *ABCG1*. Indeed, SCGB2A2 (also referred to as *MGB1)* is a secretoglobin protein expression of which is highest in the secretory phase, and increases in response to hormonal stimulation[39]. ABCG1 is a cholesterol transporter present in glandular structures of mammary tissues[40].

- The **Glandular secretory** population expresses *PAEP*, *DPP4* and *GPX3*. PAEP is a glycoprotein characteristic of the decidualised receptive endometrium[41]. The expression of the enzymes DPP4 and GPX3 has been previously reported to increase during the window of implantation[42]. Additionally, these genes are part of six genes that can be used together to reliably estimate the timing of the menstrual cycle stage of endometrial tissues[43].

- Finally, in the late secretory phase, the **Glandular secretory (*FGF7*+)** population expressed *PTPRR*, *FGF7* and *FXYD*. PTPRR has been previously shown to participate in insulin signalling [44], and its expression in the endometrium has previously been shown to be highest in the secretory phase[45]. FGF7 has previously been reported to play a role in the morphogenesis of epithelium and

re-epithelialization of wounds, and we can speculate that it may play a similar role in the re-epithelialization of the endometrial epithelium[46].

Luminal cells

During the secretory phase, in the luminal epithelium we identify preLuminal and Luminal populations. The SOX9 luminal (*LGR5*+) population is detected during the proliferative phase and is discussed in detail in the SOX9+ section above.

- The **preLuminal** cells appear during the early secretory phase of the menstrual cycle, and are characterised by the expression of *VTCN1*, *CLDN22*, *SULT1E1*. To a lesser extent, they also express markers of the previously reported luminal population (*LEFTY1*, *LGR5*, *PTGS1*, *IL6*). The most specific marker, *SULT1E1*, encodes an oestrogen sulfotransferase. *SULT1E1* expression is higher in the secretory than the proliferative phase, and inactivation of oestrogen by SULT1E1 is an important component of the temporal regulation of oestrogen bioactivity [47].

- By the mid-secretory phase, the luminal epithelium is dominated by the previously reported **Luminal** cells[2]. These cells express genes involved in TGFβ signalling (*LEFTY1*, *LGR5*), and genes important for endometrial receptivity (*PTSG1*, *IL6*). LEFTY1 is an inhibitor of the TGFβ signalling pathway, expression of which has been shown to increase from proliferative to secretory phase[48] and the mouse homolog has previously been shown to participate in endometrial remodelling through regulation of matrix metalloproteinases[49]. PTSG1 is a cyclo-oxygenase enzyme representing a rate limiting step for prostaglandin synthesis, which in turns plays an important role in embryo implantation[50]. IL-6 is an inflammatory cytokine which plays an important role in pregnancy and is decreased in patients with Recurrent Implantation Failure (RIF) (reviewed in[51]).

Ciliated cells

The ciliated cells can be divided into preCiliated cells (*CCNO, CDC20B, MUC12, PIFO, FOXJ1, TP73*) and Ciliated cells (*PIFO, FOXJ1, TP73*). TP73 and FOXJ1 are key transcription factors initiating programmes of ciliogenesis (reviewed in[52]). PIFO is involved in cilia disassembly[53].

The **preCiliated** cells were described in[2] and express the transmembrane mucin gene *MUC12*, which may play a role in epithelial barrier formation and interaction with host microbiota at mucosal surfaces (reviewed in[54]). *CCNO* and *CDC20B* are cell cycle genes involved in ciliogenesis, in part through their role in centriole amplification and deuterostomes formation for cilia assembly (reviewed in[52]). Mutations in *CCNO* are associated with chronic destructive lung disease due to reduced number of multiple motile cilia on the epithelial surface[55].

MUC5B cells in endocervix/cervix and *MUC5B* gene expression in endometrium

We identify a population of **MUC5B cells** characterised by the expression of *BPIFB1*, *TFF3*, *MUC5B*, *SAA1*. This population shows transcriptional similarity to endocervical columnar epithelial cells when projecting a publicly available scRNA-seq dataset of the cervix[56] onto our HECA **(Extended Data Fig. 1h)**. *MUC5B* and *TFF3* are known markers of cervical columnar epithelium[56]: *TFF3* is found in the surface epithelium of the endocervix, in cervical epithelium glandular structures and cervical mucus[57] and *MUC5B* has been previously used as an endocervix-specific marker[58]. We found the MUC5B cell population in biopsies from endometrial pipelle biopsies. When staining full-thickness endometrial biopsies using smFISH, we detected varied expression of *MUC5B* gene between samples, suggesting the gene is also present and expressed by endometrial cells (**Extended Data Fig. 7**). As the endometrium is a mucosal tissue known to secrete mucins, especially during the secretory phase, the detection of more wide-spread *MUC5B* smFISH signal in full-thickness endometrial biopsies of a secretory phase sample is in line with previous findings[59]. Further work is now required to demonstrate and help resolve the heterogeneity of the MUC5B cell type, and its spatial regionalisation along the reproductive tract, from cervix to endometrium.

KRT5 cells

We report a KRT5 population characterised by the expression of *TP63*, *SNCG* and *KRT5*. *KRT5* and *TP63* are markers of squamous cervical epithelium[56], and have been previously used to characterise endocervical epithelial subtypes[60]. This population is present in the cervix which in our case is present due to the inherent cell contamination that occurs as a result of sampling the endometrium by passing a Pipelle sampler through the vaginal canal and cervix first.

Supplementary Note 4. Annotation of the stromal cells.

To annotate the stromal cell states present in the human endometrium, we used the same approach described in Supplementary Note 3 for the annotation of epithelial cells which considered: (i) the distinctive expression of genes, including bona fide markers (**Fig. 3a**), (ii) the menstrual stage at which these cells appear (**Fig. 1f**), and (iii) their spatial coordinates, as inferred by integrating single-cell transcriptomics with Spatial Transcriptomics (Visium) (**Fig. 3b**). See **Methods** section "Annotation of cell types" for further information. Altogether, we resolved the following stromal cell states:

Cell type	Cell state	Markers and distinctive genes	Menstrual stages	Location
Stromal	eStromal MMPs	*MMP10, INHBA, COL8A1, MMP3, MMP1, F13A1, TGFB1, FOXL2, CRABP2, MMP11*	Proliferative	Functionalis stroma
Stromal	eStromal	*TUNAR, F13A1, TGFB1, FOXL2, CRABP2, MMP11*	Proliferative	Functionalis stroma
Stromal	eStromal cycling	*TUNAR, F13A1, TGFB1, FOXL2, CRABP2, MMP11*	Proliferative	Functionalis stroma
Stromal	dStromal early	*PLCL1, PCSK6, TPPP3, CILP, CALB2*	Secretory - Early	Functionalis stroma
Stromal	dStromal mid	*TLR4, CXCL13, SCARA5, GABRA2, DKK1, LMCD1, IL15, C3, PALMD, CFD*	Secretory - Mid	Functionalis stroma
Stromal	dStromal late	*CXCL2, LEFTY2, TRIB1, CXCL8, SMAD7, DKK1, LMCD1, IL15, C3, PALMD, CFD*	Secretory - Late	Functionalis stroma

Within the *functionalis* layer, endometrial stromal cells (eStromal) specific to the proliferative phase and decidualised stromal cells (dStromal) specific to the secretory phase were defined previously at the single-cell level[2,61]. In the HECA, we identified further heterogeneity within these two broad groups of eStromal and dStromal cells, which are described in detail below.

<u>eStromal cells</u>

- <u>eStromal MMPs:</u> For the first time, we identify a sub-population of stroma cells expressing *INHBA*, *COL8A1*, *MMP3*, *MMP1*, and *MMP10* present predominantly in samples from the menstrual phase. Matrix metallo-proteinases are involved in extracellular matrix remodelling, and thus play a critical role in endometrial remodelling during and after menstruation (reviewed in [62]).

- <u>eStromal:</u> eStromal cells were characterised by the expression of *TUNAR*, *F13A1*, *TGFB1*, *FOXL2*, *CRABP2*, *MMP11* and presence in proliferative phase biopsies. *TUNAR* is a long non-coding RNA, whose overexpression has been described to

inhibit decidualization of endometrial stromal cells[63]. *F13A1* encodes coagulation factor XIII A subunit, which is activated in the final step of the blood coagulation cascade. Mutations in *F13A1* are associated with recurrent spontaneous abortion[64]. FOXL2 is a transcription factor whose expression has been shown to negatively impact endometrial receptivity in mice[65]. CRABP2 (cellular retinoic acid-binding protein 2) was expressed in the stroma during the proliferative but not secretory phase, consistent with previous reports of *CRABP2* downregulation during decidualization[66].

- eStromal cycling: This is a cell population actively cycling, undergoing cell divisions.

dStromal cells

- dStromal early: The decidualised stromal early cells were characterised by the expression of *PLCL1*, *PCSK6*, *TPPP3*, *CILP*, *CALB2* and presence in early-secretory phase biopsies. *PLCL1* was previously reported to be expressed in decidualising stromal cells in response to progesterone[67]. *TPPP3* may also be required for decidualisation, as inhibition impairs this process due to reduced β-catenin/NF-κB/COX-2 signalling[68], and expression is downregulated in patients with RIF[69]. Studies in mice showed that *PCSK6* is important for implantation and stromal cell decidualisation[70,71]. In humans, in vitro analyses of endometrial stromal cells showed that in order for *PCSK6* expression to increase, the cells needed to be stimulated with both oestrogen and progesterone and without *PCSK6*, the stromal cells would not become decidualised[72].

- dStromal mid: The decidualised stromal mid cells were characterised by expression of *TLR4*, *CXCL13*, *SCARA5*, *GABRA2* and presence in mid-secretory phase biopsies. TLR4 is a receptor involved in innate immunity which was previously reported to be expressed in stromal cells[73] and a key molecule in the response to seminal fluid for conception in mice[74]. The chemokine CXCL13 has also been reported to be involved in embryo implantation, with endometrial expression highest in the secretory phase[75]. *SCARA5* is a marker of stromal decidualisation[76].

- dStromal late: The decidualised stromal late cells were characterised by the expression of *CXCL2*, *LEFTY2*, *TRIB1*, *CXCL8*, *SMAD7* and presence in late-secretory phase biopsies. *LEFTY2* is a premenstrual marker[77]. LEFTY2 and SMAD7 inhibit the TGFβ signalling pathway, thus providing temporal regulation of TGFβ signalling in the endometrium (see **Main Text**). CXCL8 has previously been shown to affect matrix metalloproteinase activity in stromal cells[78].

Supplementary Note 5. Differential cell abundance (DCA).

To quantify changes in cellular composition, we used differential abundance analysis on cell neighbourhoods with RMilo v1.6.0[79].

To evaluate compositional changes of immune cells between the proliferative or the secretory phases of the menstrual cycle, we first calculated the KNN graph derived from the scVI immune-embedding subsetted to contain only superficial biopsies from controls (excluding donors taking exogenous hormones). Next, we assigned cells to neighbourhoods and counted the number of cells belonging to each cell type in each neighbourhood. We assigned each neighbourhood to a cell type label based on majority voting of the cells belonging to that neighbourhood. Cell neighbourhoods where less than 70% of cells came from a single cell type were labelled as “Mixed neighbourhoods” and discarded. To test for differential abundance across the menstrual cycle, we divided the samples into proliferative and the secretory phases. RMilo models the cell count in neighbourhoods as a negative binomial generalised linear model, using a log-linear model to model the effects of menstrual phase on cell counts, while accounting for the total number of cells over all the neighbourhoods. When analysing the scRNA-seq dataset, we also included the study id as covariate of the model to account for the variability between laboratory/technical batches. A neighbourhood was associated with the proliferative phase if SpatialFDR < 0.1 and logFC < 0, or the secretory phase if SpatialFDR < 0.1, logFC > 0. The analysis was performed primarily with the snRNA-seq datasets to minimise laboratory bias, and validated on the scRNA-seq dataset where we included the study id as model covariate.

To evaluate compositional changes of mesenchymal, epithelial, endothelial and immune cells between endometriosis and controls, we again relied on the KNN graph derived from the scVI lineage-subanalysis embedding. Nuclei from donors under exogenous hormone therapy were excluded. Following the strategy described above, we estimated differential abundance between control and endometriosis case samples using the snRNA-seq dataset, as it has an even coverage of cases and controls along the menstrual cycle and avoids laboratory bias. Stromal and epithelial populations are menstrual-phase specific, and were tested considering donors in the corresponding phase. For testing differences in immune cells we instead added the menstrual phase as a covariate in the model. A neighbourhood was associated with control samples if SpatialFDR < 0.1 and logFC < 0, or endometriosis case samples if SpatialFDR < 0.1, logFC > 0.

Supplementary Note 6. Cell type enrichment analysis for Endometriosis-GWAS genes.

To study the association between the endometrial cell populations in our atlas and previously identified endometriosis GWAS loci, we used the functional GWAS (fGWAS) approach described in[80]. This approach evaluates the enrichment of various functional annotations for molecular quantitative traits (in this case, the gene expression signature of a cell type) and GWAS loci (in this case, the cis-regulatory variants associated with endometriosis). Here, genetic variants were linked to genes if they map to their cis-regulatory region, which is defined as ±500 Kb centred at the transcription start site (TSS) of the gene. The association statistics (the log odds ratios and standard errors) were transformed into the approximate Bayes factors using the Wakefield method[81,82]. The Bayes factors of variants mapping to each gene cis-regulatory region were weighted and averaged by the prior probability, estimated as the exponential function to TSS proximity. Finally, the enrichment of each cell type was estimated as the maximum likelihood estimator of the effect size for the cell-type-specific expression.

Endometriosis GWAS loci were derived from the full summary statistics of our recent endometriosis GWAS meta-analysis[83,84] excluding the *23andMe* dataset. The full summary statistics, indicating the SNP position, beta value and standard error used to perform the fGWAS analysis, are publicly available from EBI GWAS Catalog (GCST90205183).

Supplementary Note 7. Differential gene expression endometriosis vs controls.

We evaluated the magnitude and significance of the differences in gene expression between endometriosis patients and controls using limma v.3.54.2. First, to avoid unwanted confounding effects, we subsetted the data to contain only superficial biopsies and excluded donors under exogenous hormonal therapy. Secondly, to account for within-sample correlations (i.e. cells coming from the same donor), pseudobulking with sum aggregation was performed prior to applying limma. Briefly, we generated 3 pseudobulks per donor and per cell type by aggregating the cells of each cell type and taking the mean gene expression within the cell type. Finally, we tested for differential expression between conditions (endometriosis vs control) using the limma-voom approach. The analysis was performed on the scRNA-seq datasets, and we reported as differentially expressed genes with FDR < 0.1.

Supplementary Note 8. Tissue processing and cryopreservation.

8.1. Tissue cryopreservation

Fresh tissue was cut into <1 mm^3 segments before being resuspended with 1 ml of ice cold Cryostor solution (CS10) (C2874-Sigma). The tissue was frozen at -80°C decreasing the temperature approximately 1°C per minute. Detailed protocol available at https://www.protocols.io/view/tissue-freezing-in-cryostor-solution-processing-bgsnjwde.

8.2. Tissue dissociation for single-cell RNA-sequencing

Cryopreserved samples were thawed at 37°C, quickly transferred to a 15 ml tube and topped-up with 13 ml of ice cold RPMI/FBS. Samples were centrifuged (500 x g, 5 min, 4°C) and the supernatant discarded. The tissue was enzymatically digested on a MACSMix rotator (set to 16 rpm speed) at 37°C in pre-warmed RPMI/FBS containing Collagenase V (Sigma-Aldrich, C9263), and DNAse I (Roche, 11284932001) with final concentrations of 1 mg/ml and 0.1 mg/ml, respectively. Digested tissue was centrifuged (500 x g, 5 min), resuspended in 10 ml of PBS and passed through a 40 μm cell strainer (BD Biosciences, 352340), generating the collagenase fraction, enriched in stromal and immune cells (Fig. 3.1). The filter was back-washed with PBS into a 50 ml tube and centrifuged (500 x g, 5 min). Supernatant was discarded and any undigested tissue within the pellet was incubated with 0.25% (v/v) trypsin-EDTA (Sigma-Aldrich, T3924) and DNAse I (0.1 mg/ml) at 37°C for 15 min on a MACSMix rotator. The digestion process was stopped by adding RPMI/FBS and samples centrifuged (500 x g, 5 min). This step yielded the trypsin fraction. The collagenase fraction was centrifuged (500 x g, 5 min) and resuspended in 2 ml of red-blood-cell (RBC) lysis buffer (eBioscience, 00-4300) for 5-10 min at room temperature. After incubation, the samples were centrifuged (500 x g, 5 min), the RBC buffer discarded and both fractions (collagenase and trypsin) resuspended in 0.04% bovine serum albumin (BSA) (Sigma-Aldrich, A9418) in PBS (v/v). The generated single-cell suspensions were stored on ice and counted before being loaded separately onto the 10x Chromium chip.

In the case of two samples (donor IDs: FX1125 and FX1176), cells from the collagenase fraction were live/dead sorted prior to loading to enrich for live cells as the cellular viability was ~50-60% for these samples. The nuclear stain DAPI (4′,6-diamidino-2-phenylindole) was used to visualise and distinguish live/dead cells and debris.

8.3. Tissue dissociation for single-nucleus RNA-sequencing

Snap-frozen endometrial pipelle biopsies were removed from cryovials and embedded in OCT for cryosectioning, storing them at -80°C overnight. The following day, the OCT blocks were left inside the cryostat for ~1 h to equilibrate to the chamber temperature of -20°C. The blocks were trimmed until reaching the tissue, when the first 10 μm thick sections for morphological assessment under a light microscope started to be collected. Three sections were placed on SuperFrost® Plus slides (ThermoFisher, 12312148) before cutting and collecting 50 μm thick sections for nuclei extraction. Depending on tissue size, between 10 to 20 sections were placed into a 7 ml Dounce tissue grinder (Sigma-Aldrich, D9063-1SET) on dry-ice and a further three 10 μm thick sections were placed on slides and stored at -80°C for later histological staining.

Tissue collected in the Dounce tissue grinder was placed on ice inside a class II safety cabinet and incubated with 3 ml of homogenisation buffer (see **Supplementary Table 7** for buffer composition) for 5 min. To help dissolve the OCT, the suspension was gently mixed with a 2 ml aspiration pipette half-way through the incubation. The tissue was then homogenised by 10-20 strokes of both pestle A and B. The number of strokes was sample-dependent - homogenisation with each pestle was performed until no resistance and tissue changes were observed. Each pestle was washed with 500 µl of the homogenisation buffer and the homogenate filtered through a 40 µm cell strainer into a new 50 ml tube. The sample was then centrifuged using the following setting: 500 x g, 6 min, 4°C, acceleration set at 0 and deceleration set to 3. After removing the supernatant, 500 µl of wash buffer (see **Supplementary Table 8** for buffer composition) was added to the cell pellet and incubated for 2 min on ice. The nuclei pellet was gently resuspended using wide-bore tips to avoid damaging the nuclei, and the yield checked using a haemocytometer and trypan blue. Next, the nuclei suspension was transferred to a 1.5 ml tube and washed twice by adding 1 ml of the wash buffer and centrifugation (500 x g, 3 min, 4°C). The supernatant was removed and nuclei resuspended in 200 µl of the wash buffer (volume was nuclei yield-dependent). To remove debris and clumps, the nuclei suspension was filtered twice through the 40 µm Flowmi® cell strainers and nuclei counted using a haemocytometer and trypan blue. The nuclei suspension were stored on ice until loading the 10x Chromium chip.

Supplementary Note 9. Donor genotyping.

Buffy coats of 33 participants were genotyped using Illumina Global Screening Array (GSA) v3 with remaining genotypes retrieved from prior genotyping rounds using Affymetrix Precision Medicine Array (9 samples, including 5 in overlap with GSA v3), and Affymetrix Axiom (4 samples, 2 in overlap with Precision Medicine Array). Samples and variants quality was assessed using standard protocol[85]. Four samples were flagged (2 due to divergent ancestry, 2 due to low genotyping rate), two of which were re-genotyped on GSA v3. Variants passing QC (49.5% for Affymetrix arrays, 76.7% for GSA) were lifted from hg19 reference to GRCh38 using pyliftover and UCSC chain (v2013-12-31) with 99.92% success rate. The lifted SNPs were aligned to GRCh38.p13 reference using plink2[86] and exported to VCF. Stand issues arising from ambiguous plink1 source data were fixed using bcftools[87] against GRCh38.p13 reference (<5% flipped).

Supplementary References

1. Wang, W. *et al.* Single-cell transcriptomic atlas of the human endometrium during the menstrual cycle. *Nat. Med.* **26**, 1644–1653 (2020).
2. Garcia-Alonso, L. *et al.* Mapping the temporal and spatial dynamics of the human endometrium in vivo and in vitro. *Nat. Genet.* **53**, 1698–1711 (2021).
3. Tan, Y. *et al.* Single-cell analysis of endometriosis reveals a coordinated transcriptional programme driving immunotolerance and angiogenesis across eutopic and ectopic tissues. *Nat. Cell Biol.* **24**, 1306–1318 (2022).
4. Lai, Z.-Z. *et al.* Single-cell transcriptome profiling of the human endometrium of patients with recurrent implantation failure. *Theranostics* **12**, 6527–6547 (2022).
5. Fonseca, M. A. S. *et al.* Single-cell transcriptomic analysis of endometriosis. *Nat. Genet.* 1–13 (2023).
6. Huang, X. *et al.* Single-cell transcriptome analysis reveals endometrial immune microenvironment in minimal/mild endometriosis. *Clin. Exp. Immunol.* **212**, 285–295 (2023).
7. Lopez, R., Regier, J., Cole, M. B., Jordan, M. I. & Yosef, N. Deep generative modeling for single-cell transcriptomics. *Nat. Methods* **15**, 1053–1058 (2018).
8. Tan, G. C. & Yee Khong, T. Cyclic endometrium and exogenous hormone effect. in *Gynecologic and Obstetric Pathology, Volume 1* 383–408 (Springer Singapore, Singapore, 2019).
9. Deligdisch, L. Hormonal pathology of the endometrium. *Mod. Pathol.* **13**, 285–294 (2000).
10. Lotfollahi, M. *et al.* Mapping single-cell data to reference atlases by transfer learning. *Nat. Biotechnol.* **40**, 121–130 (2022).
11. Kamachi, Y. & Kondoh, H. Sox proteins: regulators of cell fate specification and differentiation. *Development* **140**, 4129–4144 (2013).
12. Danopoulos, S. *et al.* Human lung branching morphogenesis is orchestrated by the

spatiotemporal distribution of ACTA2, SOX2, and SOX9. *Am. J. Physiol. Lung Cell. Mol. Physiol.* **314**, L144–L149 (2018).

13. Blache, P. *et al.* SOX9 is an intestine crypt transcription factor, is regulated by the Wnt pathway, and represses the CDX2 and MUC2 genes. *J. Cell Biol.* **166**, 37–47 (2004).
14. Sun, D. *et al.* SOX9 maintains human foetal lung tip progenitor state by enhancing WNT and RTK signalling. *EMBO J.* **41**, e111338 (2022).
15. Mori-Akiyama, Y. *et al.* SOX9 is required for the differentiation of paneth cells in the intestinal epithelium. *Gastroenterology* **133**, 539–546 (2007).
16. Bastide, P. *et al.* Sox9 regulates cell proliferation and is required for Paneth cell differentiation in the intestinal epithelium. *J. Cell Biol.* **178**, 635–648 (2007).
17. Valentijn, A. J. *et al.* SSEA-1 isolates human endometrial basal glandular epithelial cells: phenotypic and functional characterization and implications in the pathogenesis of endometriosis. *Hum. Reprod.* **28**, 2695–2708 (2013).
18. Nguyen, H. P. T. *et al.* N-cadherin identifies human endometrial epithelial progenitor cells by in vitro stem cell assays. *Hum. Reprod.* **32**, 2254–2268 (2017).
19. Ma, S. *et al.* Expression of ALDH1A Isozymes in Human Endometrium with and without Endometriosis and in Ovarian Endometrioma. *Reprod. Sci.* **27**, 443–452 (2020).
20. Marie, P. J. & Haÿ, E. Cadherins and Wnt signalling: a functional link controlling bone formation. *Bonekey Rep* **2**, 330 (2013).
21. Loh, C.-Y. *et al.* The E-Cadherin and N-Cadherin Switch in Epithelial-to-Mesenchymal Transition: Signaling, Therapeutic Implications, and Challenges. *Cells* **8**, (2019).
22. Dwivedi, A., Slater, S. C. & George, S. J. MMP-9 and -12 cause N-cadherin shedding and thereby beta-catenin signalling and vascular smooth muscle cell proliferation. *Cardiovasc. Res.* **81**, 178–186 (2009).
23. Jho, E.-H. *et al.* Wnt/beta-catenin/Tcf signaling induces the transcription of Axin2, a negative regulator of the signaling pathway. *Mol. Cell. Biol.* **22**, 1172–1183 (2002).
24. Lustig, B. *et al.* Negative feedback loop of Wnt signaling through upregulation of conductin/axin2 in colorectal and liver tumors. *Mol. Cell. Biol.* **22**, 1184–1193 (2002).

25. Gehart, H. & Clevers, H. Tales from the crypt: new insights into intestinal stem cells. *Nat. Rev. Gastroenterol. Hepatol.* **16**, 19–34 (2019).
26. Ostrin, E. J. *et al.* β-Catenin maintains lung epithelial progenitors after lung specification. *Development* **145**, (2018).
27. Watt, F. M. Unexpected Hedgehog-Wnt interactions in epithelial differentiation. *Trends Mol. Med.* **10**, 577–580 (2004).
28. Roberson, E. C. *et al.* Hedgehog signaling is required for endometrial remodeling and myometrial homeostasis in the cycling mouse uterus. *iScience* **26**, 107993 (2023).
29. van den Brink, G. R. *et al.* Indian Hedgehog is an antagonist of Wnt signaling in colonic epithelial cell differentiation. *Nat. Genet.* **36**, 277–282 (2004).
30. Kastrati, I., Canestrari, E. & Frasor, J. PHLDA1 expression is controlled by an estrogen receptor-NFκB-miR-181 regulatory loop and is essential for formation of ER+ mammospheres. *Oncogene* **34**, 2309–2316 (2015).
31. von Maltzahn, J., Bentzinger, C. F. & Rudnicki, M. A. Wnt7a-Fzd7 signalling directly activates the Akt/mTOR anabolic growth pathway in skeletal muscle. *Nat. Cell Biol.* **14**, 186–191 (2011).
32. Chae, W.-J. & Bothwell, A. L. M. Canonical and Non-Canonical Wnt Signaling in Immune Cells. *Trends Immunol.* **39**, 830–847 (2018).
33. de Lau, W., Peng, W. C., Gros, P. & Clevers, H. The R-spondin/Lgr5/Rnf43 module: regulator of Wnt signal strength. *Genes Dev.* **28**, 305–316 (2014).
34. Carmon, K. S., Lin, Q., Gong, X., Thomas, A. & Liu, Q. LGR5 interacts and cointernalizes with Wnt receptors to modulate Wnt/β-catenin signaling. *Mol. Cell. Biol.* **32**, 2054–2064 (2012).
35. Koo, B.-K. & Clevers, H. Stem cells marked by the R-spondin receptor LGR5. *Gastroenterology* **147**, 289–302 (2014).
36. Stone, D. M. *et al.* Characterization of the human suppressor of fused, a negative regulator of the zinc-finger transcription factor Gli. *J. Cell Sci.* **112 (Pt 23)**, 4437–4448 (1999).

37. Kim, K. H. *et al.* Expression of sonic hedgehog signaling molecules in normal, hyperplastic and carcinomatous endometrium. *Pathol. Int.* **59**, 279–287 (2009).

38. Li, P., Mao, X., Ren, Y. & Liu, P. Epithelial cell polarity determinant CRB3 in cancer development. *Int. J. Biol. Sci.* **11**, 31–37 (2015).

39. Classen-Linke, I. *et al.* Mammaglobin 1: not only a breast-specific and tumour-specific marker, but also a hormone-responsive endometrial protein. *Histopathology* **61**, 955–965 (2012).

40. Mani, O. *et al.* Expression, localization, and functional model of cholesterol transporters in lactating and nonlactating mammary tissues of murine, bovine, and human origin. *Am. J. Physiol. Regul. Integr. Comp. Physiol.* **299**, R642–54 (2010).

41. Glycodelin A is expressed differentially in normal human endometrial tissue throughout the menstrual cycle as assessed by immunohistochemistry and in situ hybridization. *Fertil. Steril.* **86**, 1488–1497 (2006).

42. Burmenskaya, O. V. *et al.* Transcription profile analysis of the endometrium revealed molecular markers of the personalized 'window of implantation' during in vitro fertilization. *Gynecol. Endocrinol.* **33**, 22–27 (2017).

43. Lipecki, J. *et al.* EndoTime: non-categorical timing estimates for luteal endometrium. *Hum. Reprod.* **37**, 747–761 (2022).

44. Bektas, A., Hughes, J. N., Warram, J. H., Krolewski, A. S. & Doria, A. Type 2 diabetes locus on 12q15. Further mapping and mutation screening of two candidate genes. *Diabetes* **50**, 204–208 (2001).

45. Sherwin, J. R. A. *et al.* Global gene analysis of late secretory phase, eutopic endometrium does not provide the basis for a minimally invasive test of endometriosis. *Hum. Reprod.* **23**, 1063–1068 (2008).

46. Werner, S. & Grose, R. Regulation of wound healing by growth factors and cytokines. *Physiol. Rev.* **83**, 835–870 (2003).

47. Rubin, G. L., Harrold, A. J., Mills, J. A., Falany, C. N. & Coughtrie, M. W. Regulation of sulphotransferase expression in the endometrium during the menstrual cycle, by oral

contraceptives and during early pregnancy. *Mol. Hum. Reprod.* **5**, 995–1002 (1999).

48. Fei, W. *et al.* A functional role of LEFTY during progesterone therapy for endometrial carcinoma. *Cell Commun. Signal.* **15**, 56 (2017).
49. Tang, M., Xu, Y., Julian, J., Carson, D. & Tabibzadeh, S. Lefty is expressed in mouse endometrium in estrous cycle and peri-implantation period. *Hum. Reprod.* **20**, 872–880 (2005).
50. Marions, L. & Danielsson, K. G. Expression of cyclo-oxygenase in human endometrium during the implantation period. *Mol. Hum. Reprod.* **5**, 961–965 (1999).
51. Vilotić, A. *et al.* IL-6 and IL-8: An Overview of Their Roles in Healthy and Pathological Pregnancies. *Int. J. Mol. Sci.* **23**, (2022).
52. Lewis, M. & Stracker, T. H. Transcriptional regulation of multiciliated cell differentiation. *Semin. Cell Dev. Biol.* **110**, 51–60 (2021).
53. Kinzel, D. *et al.* Pitchfork regulates primary cilia disassembly and left-right asymmetry. *Dev. Cell* **19**, 66–77 (2010).
54. van Putten, J. P. M. & Strijbis, K. Transmembrane Mucins: Signaling Receptors at the Intersection of Inflammation and Cancer. *J. Innate Immun.* **9**, 281–299 (2017).
55. Wallmeier, J. *et al.* Mutations in CCNO result in congenital mucociliary clearance disorder with reduced generation of multiple motile cilia. *Nat. Genet.* **46**, 646–651 (2014).
56. Liu, C. *et al.* Single-cell dissection of cellular and molecular features underlying human cervical squamous cell carcinoma initiation and progression. *Sci Adv* **9**, eadd8977 (2023).
57. Wiede, A. *et al.* Synthesis and localization of the mucin-associated TFF-peptides in the human uterus. *Cell Tissue Res.* **303**, 109–115 (2001).
58. Lõhmussaar, K. *et al.* Patient-derived organoids model cervical tissue dynamics and viral oncogenesis in cervical cancer. *Cell Stem Cell* **28**, 1380–1396.e6 (2021).
59. Gipson, I. K. *et al.* Mucin Genes Expressed by Human Female Reproductive Tract Epithelia1. *Biol. Reprod.* **56**, 999–1011 (1997).

60. Chumduri, C. *et al.* Opposing Wnt signals regulate cervical squamocolumnar homeostasis and emergence of metaplasia. *Nat. Cell Biol.* **23**, 184–197 (2021).
61. Tulac, S. *et al.* Identification, characterization, and regulation of the canonical Wnt signaling pathway in human endometrium. *J. Clin. Endocrinol. Metab.* **88**, 3860–3866 (2003).
62. Curry, T. E., Jr & Osteen, K. G. Cyclic changes in the matrix metalloproteinase system in the ovary and uterus. *Biol. Reprod.* **64**, 1285–1296 (2001).
63. Wang, Y. *et al.* A Novel Molecule in Human Cyclic Endometrium: LncRNA TUNAR Is Involved in Embryo Implantation. *Front. Physiol.* **11**, 587448 (2020).
64. Xu, Z. *et al.* Polymorphisms of F2, PROC, PROZ, and F13A1 Genes are Associated With Recurrent Spontaneous Abortion in Chinese Han Women. *Clin. Appl. Thromb. Hemost.* **24**, 894–900 (2018).
65. Elbaz, M., Hadas, R., Bilezikjian, L. M. & Gershon, E. Uterine Foxl2 regulates the adherence of the Trophectoderm cells to the endometrial epithelium. *Reprod. Biol. Endocrinol.* **16**, 12 (2018).
66. Ozaki, R. *et al.* Reprogramming of the retinoic acid pathway in decidualizing human endometrial stromal cells. *PLoS One* **12**, e0173035 (2017).
67. Muter, J. *et al.* Progesterone-Dependent Induction of Phospholipase C-Related Catalytically Inactive Protein 1 (PRIP-1) in Decidualizing Human Endometrial Stromal Cells. *Endocrinology* **157**, 2883–2893 (2016).
68. Shukla, V., Kaushal, J. B., Sankhwar, P., Manohar, M. & Dwivedi, A. Inhibition of TPPP3 attenuates β-catenin/NF-κB/COX-2 signaling in endometrial stromal cells and impairs decidualization. *J. Endocrinol.* **240**, 417–429 (2019).
69. Yang, J. *et al.* Endometrial proteomic profile of patients with repeated implantation failure. *Front. Endocrinol.* **14**, 1144393 (2023).
70. Nie, G. *et al.* Inhibiting uterine PC6 blocks embryo implantation: an obligatory role for a proprotein convertase in fertility. *Biol. Reprod.* **72**, 1029–1036 (2005).
71. Nie, G.-Y., Li, Y., Minoura, H., Findlay, J. K. & Salamonsen, L. A. Specific and transient

up-regulation of proprotein convertase 6 at the site of embryo implantation and identification of a unique transcript in mouse uterus during early pregnancy. *Biol. Reprod.* **68**, 439–447 (2003).

72. Okada, H., Nie, G. & Salamonsen, L. A. Requirement for proprotein convertase 5/6 during decidualization of human endometrial stromal cells in vitro. *J. Clin. Endocrinol. Metab.* **90**, 1028–1034 (2005).
73. Guo, J. *et al.* LPS/TLR4-mediated stromal cells acquire an invasive phenotype and are implicated in the pathogenesis of adenomyosis. *Sci. Rep.* **6**, 21416 (2016).
74. Schjenken, J. E., Glynn, D. J., Sharkey, D. J. & Robertson, S. A. TLR4 Signaling Is a Major Mediator of the Female Tract Response to Seminal Fluid in Mice. *Biol. Reprod.* **93**, 68 (2015).
75. Franasiak, J. M. *et al.* Endometrial CXCL13 expression is cycle regulated in humans and aberrantly expressed in humans and Rhesus macaques with endometriosis. *Reprod. Sci.* **22**, 442–451 (2015).
76. Lucas, E. S. *et al.* Recurrent pregnancy loss is associated with a pro-senescent decidual response during the peri-implantation window. *Commun Biol* **3**, 37 (2020).
77. Tang, M., Naidu, D., Hearing, P., Handwerger, S. & Tabibzadeh, S. LEFTY, a member of the transforming growth factor-beta superfamily, inhibits uterine stromal cell differentiation: a novel autocrine role. *Endocrinology* **151**, 1320–1330 (2010).
78. Mulayim, N., Savlu, A., Guzeloglu-Kayisli, O., Kayisli, U. A. & Arici, A. Regulation of endometrial stromal cell matrix metalloproteinase activity and invasiveness by interleukin-8. *Fertil. Steril.* **81 Suppl 1**, 904–911 (2004).
79. Dann, E., Henderson, N. C., Teichmann, S. A., Morgan, M. D. & Marioni, J. C. Differential abundance testing on single-cell data using k-nearest neighbor graphs. *Nat. Biotechnol.* **40**, 245–253 (2022).
80. Elmentaite, R. *et al.* Cells of the human intestinal tract mapped across space and time. *Nature* **597**, 250–255 (2021).
81. Elmentaite, R. *et al.* Cells of the human intestinal tract mapped across space and time.

Nature **597**, 250–255 (2021).

82. Wakefield, J. A Bayesian measure of the probability of false discovery in genetic epidemiology studies. *Am. J. Hum. Genet.* **81**, 208–227 (2007).
83. D'Hooghe, T. *Biomarkers for Endometriosis: State of the Art*. (Springer, 2017).
84. Rahmioglu, N. *et al.* The genetic basis of endometriosis and comorbidity with other pain and inflammatory conditions. *Nat. Genet.* **55**, 423–436 (2023).
85. Anderson, C. A. *et al.* Data quality control in genetic case-control association studies. *Nat. Protoc.* **5**, 1564–1573 (2010).
86. Chang, C. C. *et al.* Second-generation PLINK: rising to the challenge of larger and richer datasets. *Gigascience* **4**, s13742–015–0047–8 (2015).
87. Danecek, P. *et al.* Twelve years of SAMtools and BCFtools. *Gigascience* **10**, (2021).
