# Supplementary material for: An integrated single-cell reference atlas of the human endometrium
Source: Nat Genet. 2024 Aug 28;56(9):1925–37. doi: 10.1038/s41588-024-01873-w (PMC11387200; doi:10.1038/s41588-024-01873-w)
Supplement: Supplementary file 2 — Reporting Summary [file 41588_2024_1873_MOESM2_ESM.pdf]

nature portfolio

Corresponding author(s): Roser Vento-Tormo

Last updated by author(s): May 15, 2024

Reporting Summary

Nature Portfolio wishes to improve the reproducibility of the work that we publish. This form provides structure for consistency and transparency in reporting. For further information on Nature Portfolio policies, see our Editorial Policies and the Editorial Policy Checklist.

Statistics

For all statistical analyses, confirm that the following items are present in the figure legend, table legend, main text, or Methods section.

n/a	Confirmed	
☐	☒	The exact sample size (*n*) for each experimental group/condition, given as a discrete number and unit of measurement
☒	☐	A statement on whether measurements were taken from distinct samples or whether the same sample was measured repeatedly
☐	☒	The statistical test(s) used AND whether they are one- or two-sided<br>*Only common tests should be described solely by name; describe more complex techniques in the Methods section.*
☐	☒	A description of all covariates tested
☐	☒	A description of any assumptions or corrections, such as tests of normality and adjustment for multiple comparisons
☐	☒	A full description of the statistical parameters including central tendency (e.g. means) or other basic estimates (e.g. regression coefficient) AND variation (e.g. standard deviation) or associated estimates of uncertainty (e.g. confidence intervals)
☐	☒	For null hypothesis testing, the test statistic (e.g. *F*, *t*, *r*) with confidence intervals, effect sizes, degrees of freedom and *P* value noted<br>*Give P values as exact values whenever suitable.*
☐	☒	For Bayesian analysis, information on the choice of priors and Markov chain Monte Carlo settings
☐	☒	For hierarchical and complex designs, identification of the appropriate level for tests and full reporting of outcomes
☒	☐	Estimates of effect sizes (e.g. Cohen's *d*, Pearson's *r*), indicating how they were calculated

Our web collection on statistics for biologists contains articles on many of the points above.

Software and code

Policy information about availability of computer code

Data collection	No software was used for data collection
Data analysis	Data analysis:<br>o Alignment, quantification and donor deconvolution of scRNA-seq and snRNA-seq data: Cell Ranger Software v.6.0.2; vireoSNP v.0.5.8.<br>o Alignment, quantification and quality control of Visium data: Space Ranger Software v.2.0.1; Scanpy v.1.7.0.<br>o Downstream scRNA-seq/snRNA-seq analysis: Scanpy v.1.7.0; Scrublet v.0.2.1 ; scVI v.0.6.8; cell2location v.0.06-alpha, scarches v.0.5.9; cellphonedb v.4.0.0; celltypist v.0.1.9; R celda v.1.6.1; R Seurat v.3; R limma v.3.54.2; ; RMilo v1.6.0; R SoupX v.1.5.0.<br>o Custom code available at https://github.com/ventolab/HECA-Human-Endometrial-Cell-Atlas.<br>o scArches tutorials at https://github.com/ventolab/HECA-Human-Endometrial-Cell-Atlas/blob/main/tutorials/query_to_ref_mapping.ipynb to support mapping new samples to the HECA reference cells based on any input gene expression count matrix.<br>o Interactive cell-cell communication visualisation platform, cellxgene objects for both scRNA-seq and snRNA-seq data and scVI model weigths for the scArches tutorial available at: https://www.reproductivecellatlas.org/endometrium_reference.html

For manuscripts utilizing custom algorithms or software that are central to the research but not yet described in published literature, software must be made available to editors and reviewers. We strongly encourage code deposition in a community repository (e.g. GitHub). See the Nature Portfolio guidelines for submitting code & software for further information.

Data

Policy information about availability of data

All manuscripts must include a data availability statement. This statement should provide the following information, where applicable:

- Accession codes, unique identifiers, or web links for publicly available datasets
- A description of any restrictions on data availability
- For clinical datasets or third party data, please ensure that the statement adheres to our policy

Datasets are available from ArrayExpress (www.ebi.ac.uk/arrayexpress), with accession number E-MTAB-14039 (sc/snRNA-seq) and E-MTAB-14058 (Visium spatial transcriptomics). Multiplexed smFISH images are available from BioStudies(www.ebi.ac.uk/biostudies), with accession number S-BIAD1182. All data is public access. Source data are provided with this paper. scRNA-seq and snRNA-seq datasets to reproduce UMAPs and dotplots can be accessed and downloaded through the web portals https://www.reproductivecellatlas.org/endometrium_reference.html.

Research involving human participants, their data, or biological material

Policy information about studies with human participants or human data. See also policy information about sex, gender (identity/presentation), and sexual orientation and race, ethnicity and racism.

Reporting on sex and gender	Information on sex and gender was not collected in the studies under which we collected samples used for our study. This information was also not provided by the previously published datasets that we re-analysed. All participants were referred to as women.
Reporting on race, ethnicity, or other socially relevant groupings	We did not report on race, ethnicity, or other socially relevant groupings as this information was not available for all participants included in the datasets analysed.
Population characteristics	Only individuals during their reproductive years were recruited and only considered having 'natural cycles' if they had not taken any hormonal treatment at least 3 months prior to sample collection. Donors with endometrial cancer were excluded. In addition, we aimed to exclude patients with other benign uterine/endometrial pathologies (i.e. fibroids, polyps, adenomyosis,hyperplasia). However, in some cases (n = 15), later histological evaluations revealed the presence of these pathologies (details can be found in Supplementary Table 1). Patients taking part in the ENDOX and FENOX studies (n = 69) were undergoing laparoscopic surgery for suspected endometriosis or infertility reasons. At the beginning of surgery, a superficial pipelle biopsy of the endometrium was taken and the presence/absence of endometriosis, including endometriosis stage (rASRM stages I-IV) assigned upon surgical evaluation during the laparoscopy. Four additional control samples (i.e. samples from donors without endometriosis) came from the Sanger Cell Atlasing Project study (n = 3) and Immunology of Subfertility study (n = 1). Absence of endometriosis was determined based on the clinical and medical history of the patients. For the Sanger Cell Atlasing Project, patients attended a coil clinic for contraceptive reasons. During the coil insertion procedure, a biopsy of the endometrium was taken in an outpatient setting. For the Immunology and Subfertility study, patients were undergoing in vitro fertilisation and an endometrial biopsy was taken in an outpatient setting one cycle before the patient became pregnant and had a live birth.
Recruitment	All tissue samples used for this study were obtained with written informed consent from all participants in accordance with the guidelines in The Declaration of Helsinki 2000. For the full-thickness uterine wall samples coming from deceased transplant organ donors, full informed consent was obtained from the donor families.
Ethics oversight	The collected superficial endometrial samples came from four studies: (i) Endometriosis Oxford (ENDOX), (ii) Fibroids and Endometriosis Oxford (FENOX), (iii) Sanger Human Cell Atlasing Project, and (iv) Immunology and Subfertility study. Both ENDOX (REC: 09/H0604/58) and FENOX (REC: 17/SC/0664) obtained ethical approvals from the Central University Research Ethics Committee, University of Oxford. Yorkshire & The Humber - Leeds East Research Ethics Committee approved the Sanger Human Cell Atlasing Project (REC: 19/YH/0441). The Immunology of Subfertility study (REC: 08/H0606/94) was approved by the Oxford Research Ethics Committee C. The collection of full-thickness uterine wall samples was approved by East of England–Cambridge South Research Ethics Committee (REC: 15/EE/0152).

Note that full information on the approval of the study protocol must also be provided in the manuscript.

Field-specific reporting

Please select the one below that is the best fit for your research. If you are not sure, read the appropriate sections before making your selection.

☒ Life sciences ☐ Behavioural & social sciences ☐ Ecological, evolutionary & environmental sciences

For a reference copy of the document with all sections, see nature.com/documents/nr-reporting-summary-flat.pdf

Life sciences study design

All studies must disclose on these points even when the disclosure is negative.

Sample size	In total, we collected samples from 75 individuals. We obtained superficial endometrial biopsies from 73 individuals and one full-thickness uterine wall sample from 1 individual (A70) for scRNA-seq and snRNA-seq experiments. We obtained an additional full-thickness uterine wall

sample (donor A66) for imaging analyses.

We generated new scRNA-seq data for 16 donors, and snRNA-seq data for 63 donors. For 5 donors, both scRNA-seq and snRNA-seq data were generated (see Replication below).

We integrated our scRNA-seq dataset (n = 16 donors) with previously published data (n = 49 donors) from the following 6 studies:

(i) Wang et al. (GEO accession number GSE111976), re-analysed 10 samples with the following donor IDs: SAMN15049042, SAMN15049043, SAMN15049044, SAMN15049045, SAMN15049046, SAMN15049047, SAMN15049048, SAMN15049049, SAMN15049050, SAMN15049051.

(ii) Garcia-Alonso et al. (ArrayExpress accession number EMTAB-10287), re-analysed 5 samples with the following donor IDs: A13, A30, E1, E2, E3.

(iii) Tan et al. (GEO accession number GSE179640), re-analysed 12 samples with the following donor IDs: C01, C02, C03, E01, E02, E03, E04, E05, E06, E07, E08, E09.

(iv) Lai et al. (GEO accession number GSE183837), re-analysed 3 samples with the following donor IDs: GSM5572238, GSM5572239, GSM5572240.

(v) Fonseca et al. GEO accession number GSE213216), re-analysed 7 samples with the following donor IDs: Fonseca_10, Fonseca_11, Fonseca_13, Fonseca_14, Fonseca_16, Fonseca_17, Fonseca_18, Fonseca_19.

(vi) Huang et al. (GEO accession number GSE214411), re-analysed 10 samples with the following donor IDs: GSM6605431, GSM6605432, GSM6605433, GSM6605434, GSM6605435, GSM6605436, GSM6605437, GSM6605438, GSM6605439, GSM6605440.

Our study analysed the largest number of individuals and cells with respect to single-cell RNA transcriptomic profiling of the endometrium. This samples set should be sufficient to capture the main cell types and states in the tissue of the menstrual cycle phases analysed.

In addition, novel subsets defined transcriptomically in our dataset (e.g. preGlandular, preLuminal, SOX9 basalis CDH2+ populations) have been validated using orthogonal methods (e.g. spatial transcriptomics, single molecule fluorescence in situ hybridisation imaging).

Data exclusions

No data were excluded from the analyses.

Replication

For 5 donors, both single-cell and single-nuclei RNA-sequencing data was generated (donor IDs: FX1119, FX1146, FX1156, FX9006). We confirmed the same cell populations could be identified in single-cell and single-nuclei data.

All smFISH experiments were replicated and validated by at least n=2 biologically independent samples.

Randomization

This is not applicable to our study as during surgery, participants were either diagnosed with endometriosis or confirmed they did not have any visible endometriosis.

Blinding

Tissue histology evaluation and menstrual phase staging of the newly collected samples was conducted by at least two independent pathologist, all of them blinded.
The rest of investigators involved in the study were aware of the clinical status of the donor and the collected metadata.

Reporting for specific materials, systems and methods

We require information from authors about some types of materials, experimental systems and methods used in many studies. Here, indicate whether each material, system or method listed is relevant to your study. If you are not sure if a list item applies to your research, read the appropriate section before selecting a response.

Materials & experimental systems

n/a	Involved in the study
☒	☐ Antibodies
☒	☐ Eukaryotic cell lines
☒	☐ Palaeontology and archaeology
☒	☐ Animals and other organisms
☒	☐ Clinical data
☒	☐ Dual use research of concern
☒	☐ Plants

Methods

n/a	Involved in the study
☒	☐ ChIP-seq
☒	☐ Flow cytometry
☒	☐ MRI-based neuroimaging
